# Preclinical Evaluation of the Efficacy of α-Difluoromethylornithine and Sulindac Against SARS-CoV-2 Infection

**DOI:** 10.3390/v17101306

**Published:** 2025-09-26

**Authors:** Natalia A. Ignatenko, Hien T. Trinh, April M. Wagner, Eugene W. Gerner, Christian Bime, Chiu-Hsieh Hsu, David G. Besselsen

**Affiliations:** 1 Department of Cellular and Molecular Medicine, The University of Arizona, Tucson, AZ 85724, USA; egerner@arizona.edu; 2 Valley Fever Center for Excellence, The University of Arizona, Tucson, AZ 85724, USA; htt@arizona.edu; 3 University Animal Care, The University of Arizona, Tucson, AZ 85724, USA; awagner@arizona.edu (A.M.W.); besselsd@arizona.edu (D.G.B.); 4 Department of Medicine, College of Medicine-Phoenix, The University of Arizona, Phoenix, AZ 85004, USA; cbime@arizona.edu; 5 Mel and Enid Zuckerman College of Public Health, The University of Arizona, Tucson, AZ 85724, USA; pchhsu@arizona.edu

**Keywords:** SARS-CoV-2, COVID-19 mouse model, antiviral drugs, polyamines, DFMO, Sulindac

## Abstract

Despite numerous research efforts and several effective vaccines and therapies developed against coronavirus disease 2019 (COVID-19), drug repurposing remains an attractive alternative approach for treatment of SARS-CoV-2 variants and other viral infections that may emerge in the future. Cellular polyamines support viral propagation and tumor growth. Here we tested the antiviral activity of two polyamine metabolism-targeting drugs, an irreversible inhibitor of polyamine biosynthesis, α-difluoromethylornithine (DFMO), and a non-steroidal anti-inflammatory drug (NSAID), Sulindac, which have been previously evaluated for colon cancer chemoprevention. The drugs were tested as single agents and in combination in the human Calu-3 lung adenocarcinoma and Caco-2 colon adenocarcinoma cell lines and the *K18-hACE2* transgenic mouse model of severe COVID-19. In the infected human cell lines, the DFMO/Sulindac combination significantly suppressed SARS-CoV-2 N1 Nucleocapsid mRNA by interacting synergistically when cells were pretreated with drugs and additively when treatment was applied to the infected cells. The Sulindac alone and DFMO/Sulindac combination treatments also suppressed the expression of the viral Spike protein and the host angiotensin-converting enzyme 2 (ACE2). In *K18-hACE2* mice, the antiviral activity of DFMO and Sulindac as single agents and in combination was tested as prophylaxis (drug supplementation started 7 days before infection) or as treatment (drug supplementation started 24 h post-infection) at the doses equivalent to patient chemoprevention trials (835 ppm DFMO and 167 ppm Sulindac). The drugs’ antiviral activity in vivo was evaluated by measuring the clinical (survival rates and clinical scores), viral (viral load and virus infectivity), and biochemical (plasma polyamine, Sulindac, and Sulindac metabolite levels) endpoints. Prophylaxis with DFMO and Sulindac as single agents significantly increased survival rates in the young male mice (*p* = 0.01 and *p* = 0.027, respectively), and the combination was effective in the aged male mice (*p* = 0.042). Young female mice benefited the most from the prophylaxis with Sulindac alone (*p* = 0.001) and the DFMO/Sulindac combination (*p* = 0.018), while aged female mice did not benefit significantly from any intervention. Treatment of SARS-CoV-2-infected animals with DFMO or/and Sulindac did not significantly improve their survival rates. Overall, our studies demonstrated that DFMO and Sulindac administration as the prophylaxis regimen provided strong protection against the lethal outcome of SARS-CoV-2 infection and that male mice benefited more from the polyamine-targeted antiviral treatment than female mice. Our findings underscore the importance of evaluation of the antiviral activity of the drugs in the context of sex and age.

## 1. Introduction

Many RNA viruses, such as influenza, Chikungunya, Zika, Ebola, and coronaviruses, present a significant danger to the human population. Coronaviruses, particularly, can impose major economic loss worldwide, which has become especially evident when the novel, highly pathogenic severe acute respiratory syndrome coronavirus 2 (SARS-CoV-2) emerged. SARS-CoV-2 caused a severe lower respiratory tract infection, named coronavirus disease 2019 (COVID-19), with acute respiratory distress and extrapulmonary organ dysfunction in infected vulnerable individuals, which resulted in a loss of more than 7 million human lives worldwide [1].

Detailed analysis of COVID-19 cases revealed significantly higher vulnerability among the older population (75 years and older) with underlying health conditions, such as diabetes, lung disease, cancer, immunodeficiency, asthma, kidney disease, and gastrointestinal disease. Additionally, males have been more susceptible to SARS-CoV-2, with a higher rate of death [2]. Even though effective vaccines and antiviral drugs specific to SARS-CoV-2 infection have been developed, there is a need to evaluate alternative prevention and treatment strategies to overcome potential resistance to existing anti-COVID-19 medications and to treat novel SARS-CoV-2 variants.

SARS-CoV-2 belongs to a family of single-stranded positive, enveloped RNA viruses, which cause respiratory, enteric, and systemic infections in animals and humans. Although SARS-CoV-2 is genetically similar to the previously evolved 2002–2003 SARS-CoV-1 and the 2012 Middle Eastern respiratory syndrome coronavirus (MERS-CoV), it has unique differences in the sequence of the external subdomain of the Spike protein receptor binding domain (RBD) and encodes two novel proteins (orf33b and orf8) [3]. SARS-CoV-2 demonstrated a much higher ability to spread due to its longer incubation period (2–14 days versus 2–7 days for SARS-CoV-1) and high infectivity during this asymptomatic period. SARS-CoV-2 infection starts with the binding of the virus to the transmembrane receptor angiotensin-converting enzyme 2 (ACE2) on the host cells, with subsequent Spike protein priming by a transmembrane protease serine 2 (TMPRSS2) [4]. Virus particles enter cells by endocytosis, and the viral RNA is translated at the endoplasmic reticulum (ER) membrane. Viral particles are transported through the ER–Golgi secretory pathway to the cell surface, where the virus is released [5]. During the viral replication, a single-stranded SARS-CoV-2 RNA initiates a cellular immune response with marked elevation in inflammatory mediators (the “cytokine storm”) [6,7].

The dependence of viruses on host cell resources for replication presents opportunities to suppress and even eliminate viral infections by targeting the host’s cellular components that viruses rely on. Notably, the importance of cellular polyamines for viral replication has been shown in numerous in vitro studies (reviewed in [8,9]). Polyamines, putrescine, spermidine, and spermine, are naturally occurring organic cations essential for the growth and development of both prokaryotic and eukaryotic cells [10,11]. Intracellular polyamine levels are tightly regulated, indicating that these molecules are critical for optimal cellular growth [12,13,14]. Polyamines participate in various cellular processes, including control of gene expression at the transcriptional, post-transcriptional, and translational levels [15,16]. Polyamines also influence the expression of pro-inflammatory genes, such as cyclooxygenase 2 (COX2) [17]. Viruses require polyamines throughout their life cycle for functions like genome packaging, DNA-dependent RNA polymerase activity, genome replication, and viral protein translation [18,19]. Spermidine and spermine, in particular, have been found in the viral envelope of herpes simplex virus and have been shown to contribute to the stability of capsid structure through interaction with the negatively charged viral DNA genome. Many viruses, including chikungunya virus, Zika virus [18], hepatitis C virus [20], herpes simplex virus [21], and even the Ebola virus [22], depend on host polyamines for viral translation. Additionally, viruses can stimulate host cell polyamine production; for instance, human cytomegalovirus induces host ODC1 activity and increases polyamine uptake in infected cells [23,24].

A synthetic irreversible inhibitor of a key biosynthetic enzyme, ornithine decarboxylase (ODC1), eflornithine (α-difluoromethylornithine or DFMO) [25] has become a valuable tool in assessing the function of polyamines in normal and diseased states. DFMO has been tested in vitro against numerous RNA viruses and has been shown to restrict the replication of flaviviruses dengue virus serotype 1, Japanese encephalitis virus, yellow fever virus, poliovirus, bunyavirus Rift Valley fever virus, and rhabdovirus rabies virus [18]. Importantly, DFMO treatment of MERS-CoV-infected Vero cells resulted in a 30-fold reduction in viral titer [18]. Inhibition of ODC1 enzyme activity by DFMO also resulted in the suppression of translation of viral transcripts due to a decrease in the cellular spermidine level, which is required for hypusination of the cellular translation factor eIF5A [22]. These findings demonstrate that DFMO antiviral activity is multimodal and can be highly effective against the SARS-CoV-2 virus. Stimulation of polyamine excretion using synthetic polyamine analogs, which induce transcription of a key polyamine catabolic enzyme spermidine/spermine N1-acetyltransferase (SAT1), has also been shown to limit the replication of RNA viruses [18].

The polyamine depletion strategy based on a combination of inhibition of polyamine biosynthesis by DFMO and activation of polyamine excretion using the non-steroidal anti-inflammatory drug (NSAID) Sulindac has been tested in colon cancer cell lines and animal models, and it has been shown that a decrease in intracellular polyamine levels leads to suppression of tumorigenesis [26,27,28]. Furthermore, DFMO in combination with Sulindac has been utilized to prevent colorectal adenomas in patients with prior colon polyps and proven to be safe and effective in preclinical studies and several clinical trials [29,30,31].

A more rigorous evaluation of drugs that target polyamine metabolism for antiviral indications is needed, specifically to ameliorate the severity of SARS-CoV-2 infection. The goal of this study was to evaluate the efficacy of well-studied drugs, such as DFMO and Sulindac, against SARS-CoV-2 infection in cell lines and in a transgenic *K18-hACE2* mouse model of severe COVID-19 disease [32]. Both DFMO and Sulindac were tested in the *K18-hACE2* mouse model at doses equivalent to those used in the completed clinical trials for colon cancer chemoprevention [30,33,34]. The results of this work provide an example of a practical alternative approach to address the need for antiviral drugs in the future.

## 2. Materials and Methods

### 2.1. Materials

All cell culture reagents used in this study were purchased from Thermo Fisher Scientific, Inc. (Waltham, MA, USA) and Sigma-Aldrich (St. Louis, MO, USA). Difluoromethylornithine hydrochloride hydrate (DFMO, product number D193) was purchased from MilliporeSigma (Burlington, MA, USA). Sulindac (cat. no. 10004386) was purchased from Cayman Chemical (Ann Arbor, MI, USA). DFMO was diluted in Dulbecco’s Modified Eagle medium (DMEM). A stock solution of Sulindac for cell culture experiments was prepared in 100% Dimethylsulfoxide (DMSO) and diluted with DMEM.

### 2.2. Cell Lines

Vero rhesus monkey kidney cell line (ATCC-CCL-81, kindly provided by Dr. Janko Nikolich), Calu-3 lung adenocarcinoma cell line (ATCC-HTB-55), and Caco-2 colon adenocarcinoma cell line (ATCC-HTB-37) were purchased from the American Type Culture Collection (ATCC) and were maintained in DMEM (4.5 g/L glucose, L-glutamine, w/o sodium pyruvate, supplemented with 10% FBS and 1% penicillin/streptomycin). Cells were incubated at 37 °C in 5% CO_2_.

### 2.3. SARS-CoV-2

All experiments involving SARS-CoV-2 were performed in compliance with the University of Arizona guidance for BSL-3 work. SARS-CoV-2 strain USA_WA1/2020 was obtained from Dr. Natalie Thornburg through the University of Texas Medical Branch (UTMB) World Reference Center for Emerging Viruses and Arboviruses (WRCEVA). The virus was propagated as previously described [35]. Briefly, 80–90% confluent Vero cells were infected with an approximate multiplicity of infection (MOI) of 0.01 and incubated for 48 h, and the supernatant was harvested and clarified. Virus titer was determined by plaque assay.

#### SARS-CoV-2 Inactivation

In cell culture experiments, the virus was inactivated using lysis buffer RAV1 (NucleoSpin RNA Virus kit; Macherey-Nagel GmbH & Co., Dueren, Germany). In animal experiments, the SARS-CoV-2 virus was inactivated in tissue lysates using lysis buffers compatible with sample processing for downstream applications, with 20× Lysis RNase Inhibitor buffer (2% Igepal CA-630 (NP40), 3 mg/mL Poly-vinylsulfonic acid (PVSA) solution in PBS) added to each sample for a final concentration of 1×. The NP-40 at a concentration of 0.1% in solution has been shown to completely inactivate SARS-CoV2 virions in lysed cells, and PVSA at a final concentration of 150 μg/mL has been shown to inhibit RNases [36,37].

### 2.4. Drug Synergy Analysis

Cell lines were seeded in 96-well plates at a concentration of 2 × 10^4^ cells per well (Vero, Caco-2 cell lines) or 3 × 10^4^ cells per well (Calu-3 cell line) in 50 μL media volume per well. For the prophylaxis regimen, 24 h after subculture, the cell culture media were replaced with media containing drugs, and cells were cultured for an additional 48 h before exposure to the virus. For the treatment regime, cells were incubated in drug-free media for 48 h before being infected. Under both regimens, cells were infected with the virus at an MOI of 0.05 for one hour, and then the virus-containing media in the wells were replaced with new media containing DFMO, Sulindac, or a combination of DFMO and Sulindac (DFMO/Sul combo). The infected control and treated cultures were harvested at 48 h post-infection. The infected cell lines were treated with the tested drugs for 72 h in duplicate. Following the virus inactivation cells were lysed with 20× Lysis RNase Inhibitor buffer added to a final concentration of 1× in a total volume of 100 μL. The contents of the wells were mixed, plates were frozen, and 5 μL of the lysed cultures were used as a template for measuring the level of viral nucleocapsid gene (*N1*) transcript by quantitative RT-PCR (qRT-PCR). The percent of *N1* inhibition by DFMO/Sulindac combo relative to the untreated control was calculated and further analyzed using a free web application SynergyFinder (https://synergyfinder.fimm.fi (accessed on 1 September 2025) [38]). The Zero Interaction Potency (ZIP) model was utilized to evaluate the potential synergy of drug combinations. Briefly, the ZIP model interprets SynergyFinder results based on the calculated summary score with a statistical significance (*p*-value) and the mean ZIP value. Mean ZIP value increases as the synergy between drugs increases, such as a score less than −10 indicates that drugs interact antagonistically, a score between −10 and 10 indicates that drugs have an additive effect, and a score >10 indicates that drugs have a synergistic effect [39].

### 2.5. Cell Culture Experiments

The antiviral activity of the tested drugs was evaluated in the Caco-2 and Calu-3 cell lines. Cells were seeded in 6-well or 24-well plates at 0.3 × 10^6^ cells/well or 0.5 × 10^5^ cells/well, respectively, in DMEM. Twenty-four hours after subculture, the cells were infected with SARS-CoV-2 at an MOI of 0.05. After 1 h, the virus-containing media were replaced with media containing the tested compounds at the following concentrations: 1 mM DFMO, 300 μM Sulindac, or DFMO/Sul combo (1 mM/300 μM), while the infected untreated cells were incubated with vehicle control (DMSO). Treatments of the infected cells were performed in triplicate. At 72 h post-infection, the virus was inactivated, and cells were lysed as described above. Samples were frozen until analyzed by qRT-PCR to quantify the drugs’ effects on the viral load and host cell gene expression.

### 2.6. Plaque Assay

The SARS-CoV-2 plaque-forming assay was performed using a liquid overlay and fixation-staining method according to the previously described protocols [35,40]. Briefly, Vero cells were plated in 24-well plates and infected with 10-fold serial dilutions of virus stock or clarified lung supernatant once cells reached 90% of confluency. Plates were incubated for 1–2 h at 37 °C in 5% CO_2_. Cell culture medium containing methylcellulose (1% *w*/*v*) was added to each well, and the plates were incubated for 72 h. Following incubation, the medium was discarded, and wells were filled with 10% formalin and incubated for 30 min to inactivate the virus and fix cells. The plates were washed with tap water, and then 0.9% Crystal Violet (*w*/*v*) in 40% ethanol solution was added to each well to stain the cells. The plates were washed and dried, and the plaques were counted to calculate titers in plaque-forming units (PFU/mL).

### 2.7. RNA Isolation

Viral RNA from the lysed cells was isolated for host cell expression analysis using the NucleoSpin RNA Virus kit (cat. no. 740956, Macherey-Nagel GmbH & Co.) according to the manufacturer’s protocol. Total RNA extraction was performed using the Qiagen RNeasyPlus Kit (cat. no. 74136, Qiagen GmbH, Hilden, Germany) according to the manufacturer’s protocol. RNA purity was assessed using a Nanodrop spectrophotometer (ND-2000; Thermo Fisher Scientific, Inc.).

### 2.8. Quantitative Reverse-Transcription PCR (qRT-PCR)

Five microliters of each lysed cell culture was used directly as a qRT-PCR template to measure the expression of the SARS-CoV-2 viral gene encoding the N1 Nucleocapsid protein. Absolute quantification of N1 was performed using SARS-CoV-2-specific in vitro-transcribed N1 RNA standard (2019-nCoV_N_Positive Control) and primers [41,42] using QuantaBio qScript XLT One-Step qRT-PCR ToughMix (cat. no. 89236-676, VWR International, Randor, PA, USA). TaqMan^®^ SARS-CoV-2 assays were performed to detect the Spike glycoprotein S1 subunit 1 (cat. no. 34221182, assay ID: V107918636-S1) and human RNase P (cat. no. 34331182, assay ID: Hs04930436-g1; ThermoFisher Scientific, Inc.), which were used for extraction and internal amplification quality control for cell culture experiments. For N1 qRT-PCR analysis in the animal experiments, 5 μL of lung tissue homogenate was used as a template, and the N1 transcript number was normalized to the expression level of the mouse glyceraldehyde-3-phosphate dehydrogenase (GAPDH) gene (cat. no. 4331182, assay ID: Mm99999915_g1) in the same sample. For the host gene expression analysis, reverse transcription was completed using the Applied Biosystems High-Capacity cDNA Reverse Transcription Kit (cat. no. 4368814, Applied Biosystems, Foster City, CA, USA). Total RNA (2 μg) was transcribed into cDNA in a 100 μL reaction using random hexamers under thermal conditions recommended by the manufacturer. The qRT-PCR was performed using human TaqMan^®^ assays specific for ornithine decarboxylase, *ODC1* (cat. no. 4331182, assay ID: Hs00159739_m1); spermine oxidase *SMOX* (assay ID: Hs00602494_m1); spermidine/spermine acetyltransferase, *SAT1* (assay ID: Hs00161511_m1); and *ACE2* (assay ID: Hs01085333_m1); The β2-microglobin (β2M, FAM (Hs99999907_m1)), gene served as an endogenous reference for the host (target) genes. Total RNA (0.2 µg) was reverse-transcribed into cDNA in a 20 µL reaction with random hexamers under thermal conditions recommended by the protocol. Real-time PCR amplification was performed using the QuantStudio 3.0 Real-Time PCR system (Applied Biosystems, Thermo Fisher Scientific, Inc. Waltham, MA, USA.) under the universal thermal cycling conditions recommended by the Assay-on-Demand products protocol. Negative controls without templates were included in each plate to monitor potential PCR contamination. The expression of genes was tested in triplicate, and each reaction was run in duplicate. The comparative *Ct* method was used to determine the relative expression level of each target gene. The *Ct* value of each target gene was normalized by the endogenous reference. The fold change in the expression of each target gene was calculated via the equation 2^−∆∆*Ct*^, where ∆Δ*C_t_* = (*C_t_*_(target)_ − *C_t_*_(endogenous control)_)_treatment_ − ((*C_t_*_(target)_ − *C_t_*_(endogenous control)_)_control_.

### 2.9. Animal Experiments

All mice were maintained in the University of Arizona’s Animal Care Facility in accordance with the University of Arizona Institutional Animal Care and Use Committee (IACUC). The animal study protocol was approved by the Institutional Animal Care and Use Committee of the University of Arizona (protocol number: 2021-0772, date of approval: 3 January 2021). The University of Arizona Animal Care facility is AAALAC-accredited and holds an Animal Welfare Assurance # D16-00159 (A-3248-01).

Six-week-old *C57BL/6J K18-hACE2* transgenic mice of both sexes were purchased from Jackson Labs (https://www.jax.org/strain/034860) (accessed on 1 September 2025). *K18-hACE2* mice express human angiotensin-converting enzyme 2 (ACE2) under the control of the promoter and first intron of the human cytokeratin 18 (K18) gene in epithelia, including airway epithelia, and recapitulate severe coronavirus disease [32]. One set of the animals was used in experiments at 6 weeks of age (young mouse groups), and another set was maintained at the facility and used in experiments upon reaching 58 weeks of age (aged mouse groups). The animals were housed in groups of five in individually ventilated microisolator cages under fluorescent lighting on a 14:10 light/dark cycle. Irradiated feed (Teklad Global diet; cat. no. 2919, Inotiv, Indianapolis, IN, USA) and hyperchlorinated reverse osmosis drinking water were available ad libitum. The animals were fed a defined synthetic diet, AIN-93G (cat. no. 3 TD.94045, Inotiv), for 7 days prior to the start of the experiments.

#### 2.9.1. Administration of Compounds

For the prophylaxis regime in 6-week-old and 58-week-old mice and the treatment regime in 58-week-old mice, DFMO at a dose of 835 ppm and Sulindac at a dose of 167 ppm were administered in custom-prepared AIN-93G diets. These dietary preparations of DFMO and Sulindac have been successfully used in murine cancer models and are based on the recommended human drug doses [29,30,43]. The treatment regime in 6-week-old mice was implemented by intragastric gavage (IG) starting at 24 h after the mice were infected with 1000 PFU of SARS-CoV-2. The drugs were administered by IG daily at 0.1 mL volume per mouse/day at doses equivalent to the doses of DFMO and Sulindac in the clinical trial for the prevention of sporadic colorectal adenomas [30]. Aqueous solutions of DFMO at 154.17 mg/kg (mg of compound per kg of body weight) in water (pH 11), Sulindac at 30.8 mg/kg in 0.25 M sodium bicarbonate, DFMO/Sulindac combo in 0.25 M sodium bicarbonate, or 0.25 M sodium bicarbonate as a vehicle control were administered. The gavage needles were pre-coated with 1 g/mL of sucrose before gavage to improve the swallowing reflex and decrease the time for passing the needle into the esophagus. Reverse-osmosis drinking water was available ad libitum for the duration of the experiments.

#### 2.9.2. Animal Infection and Monitoring

Mice were inoculated with SARS-CoV-2 virus intranasally at a dose of 1000 plaque-forming units (PFU) immediately after brief sedation with inhaled isoflurane. The standardized SARS-CoV-2 dose of 1000 PFU was selected for mouse infections based on a 20% survival rate at 14 dpi in 6-week-old *K18-hACE2* mice. Non-invasive body weight measurement and clinical symptom scoring were performed daily. Specifically, infected animals were observed daily for signs of body weight loss, rapid breathing, hunched posture, lethargy, inactivity, and/or rough hair coat. The following clinical scoring scales were used: for weight: 0, no weight loss; 1, <10% weight loss; 2, 10–19% weight loss; 3, >20% weight loss; for breathing: 0, normal; 1, mild tachypnea (mild increase in RR); 2, severe tachypnea (marked increase in RR); 3, dyspnea (deep, labored breathing); for overall status: 0, healthy; 1, less than normal activity; 2, inactive; 3, no movement; 4, no response to stimuli; 5, dead. Mice were considered moribund and euthanized if they exhibited a >4 °C drop in body temperature, >20% body weight loss, severe dyspnea, inability to ambulate, inability to access food/water, paralysis, or non-responsiveness to stimulation.

#### 2.9.3. Sample Collection and Processing

Animals were euthanized at 10–14 days post-infection using isoflurane. Blood was collected into EDTA-containing tubes and centrifuged. Plasma (25 μL) was treated with NP-40 (to inactivate virus) and frozen at 80 °C until processed for polyamine and Sulindac metabolite analysis. Left lung tissue (80–100 mg) was fixed in 4% paraformaldehyde (PFA) solution in 0.1 M phosphate buffer (ThermoFisher Scientific, Inc.) overnight at room temperature, moved to 70% ethanol, paraffin-embedded, and cut into 6 μm sections for hematoxylin and eosin (H&E) staining. Right lung tissue (80–100 mg) was homogenized with glass beads in 500 μL PBS using a Mini-Beadbeater-96 (Biospec Products, Bartlesville, OK, USA) at a maximum speed of 2400 rpm/s for 2 min following centrifugation at 5000 rpm for 10 min at room temperature. The supernatant was evaluated by plaque assay.

### 2.10. Histological Analysis of Lung Inflammation

The lung H&E slides were coded and evaluated by a board-certified veterinary pathologist. The perivascular, peribronchiolar, alveolar inflammation, alveolar edema, and airway necrosis were assessed using the following scoring system: 0, normal; 1, mild, infrequent necrosis and few inflammatory cells; 2-moderate, multifocal necrosis and thin layers of inflammation; 3, marked multifocal to segmental necrosis and thick layers of inflammation; 4, severe, coalescing necrosis and inflammation.

### 2.11. Polyamine and Sulindac Metabolite Analysis

Ten microliters of plasma samples were assayed for polyamine content by reverse-phase high-performance liquid chromatography (HPLC) with 1,7-diaminoheptane as an internal standard [44]. Amines detectable by this method included putrescine, cadaverine, histamine, spermidine, and spermine, with a limit of detection of 5 pmol. Data are expressed as nmol polyamine per ml. The HPLC analysis of Sulindac and its metabolites, Sulindac sulfide and Sulindac sulfone, was performed using plasma samples diluted 1:50. The aliquots of diluted serum were mixed with the internal standard solution (100 ng/mL of indomethacin in acetonitrile) and processed according to the previously described method [45].

### 2.12. Statistical Analysis

Statistical analysis of viral and host gene expression in cell lines and analysis of the degree of lung inflammation were performed using the ANOVA single-factor test. A *p*-value < 0.05 was considered statistically significant. Kaplan–Meier estimation was performed to derive the median survival time by treatment in different experimental groups of animals. Cox regression was performed to derive the hazard ratio (vs. control) for each treatment group. The clinical scores of animals at the time of euthanasia were summarized using means ± SDs by group. A one-way ANOVA test was performed to compare clinical scores between treatment groups. Polyamine content and Sulindac metabolite levels in plasma were summarized using means ± SDs by group. One-way ANOVA/two-sample *t*-test was performed to compare polyamine content and Sulindac metabolites between groups. The *p*-value (vs. untreated control) by sex was derived from one-way ANOVA and the interaction *p*-value between treatment and sex was derived from two-way ANOVA with the interaction terms between treatment and sex indicators.

For a change in weight analysis, the average daily percent (%) weight change during the course of the experiments was calculated for each experimental group (mean ± SD). A linear mixed-effects model with a random intercept accounting for within-subject correlation was fitted to compare the trajectory of daily % weight changes between groups after adjusting for weight at Day 0. Additionally, weight comparison was performed for the experimental groups between Day 0 and Day 4 post-infection. For each study, daily weights at Day 0 and Day 4 were summarized using means ± SDs by group. One-way ANOVA was performed to compare % weight change at Day 4 from Day 0 after adjusting for the weight at Day 0. If *p* < 0.05 for one-way ANOVA, Tukey’s HSD (Honestly Significant Difference) test was performed to detect the significant pairwise differences while controlling for multiple comparisons.

## 3. Results

### 3.1. Evaluation of the Antiviral Activity of DFMO and Sulindac In Vitro

**Figure 1:**
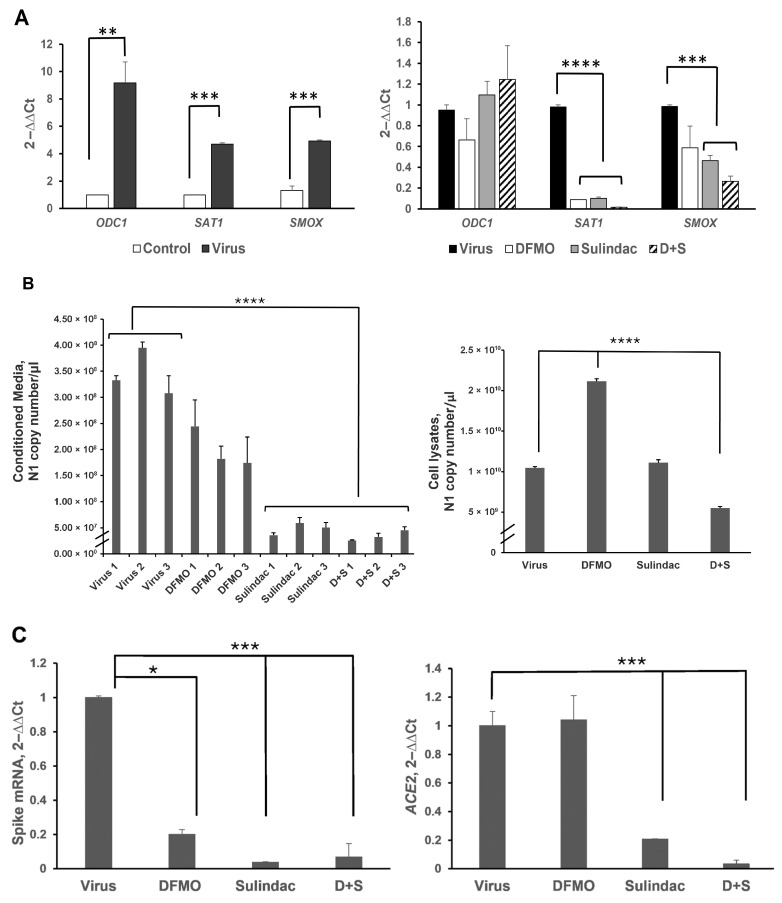
Analysis of the viral and host gene expression in the Calu-3 cell line infected with SARS-CoV-2 at 0.05 MOI and treated with 1 mM DFMO, 300 μM Sulindac, or 1 mM DFMO/ 300 μM Sulindac combination at 72 h post-infection by qRT-PCR. (**A**). (**Left panel**). The fold changes in the expression of polyamine metabolic enzymes in Calu-3 cells uninfected (Control) and infected (Virus) with SARS-CoV-2 virus at MOI 0.05. (**Right panel**). Fold changes in expression of polyamine metabolic genes in the infected Calu-3 cells due to treatment with DFMO, Sulindac, and DFMO/Sul combination for 72 h. (**B**). (**Left panel**). SARS-CoV-2 Nucleocapsid (N1) RNA copy number in the conditioned media of infected Calu-3 cells, untreated (Virus, 1–3), or treated with DFMO (DFMO, 1–3), Sulindac (Sulindac, 1–3), and DFMO/Sulindac combination (D + S, 1–3) for 72 h post-infection. The *N1* copy number was measured, and the results from three independent experiments are shown. (**Right panel**). N1 RNA copy number in cell lysates, infected untreated (Virus), DFMO-treated (DFMO), Sulindac-treated (Sulindac), and DFMO/Sulindac-treated (D + S). (**C**). (**Left panel**). Fold changes in transcript levels of Spike viral protein in Calu-3 cell lysates infected untreated (Virus) and treated with DFMO, Sulindac, or DFMO/Sulindac combination (D + S). (**Right panel**). Fold changes in expression of *ACE2* mRNA in Calu-3 cells infected untreated (Virus) and treated with compounds. RNA levels of viral genes were normalized to the levels of the human RNase P in cells. Data were analyzed using the ANOVA single-factor test. The results, representative of three independent experiments, are shown. * *p* = 0.02, ** *p* = 0.01, *** *p* < 0.003, **** *p* < 0.0001.

Our initial evaluation of the antiviral activity of DFMO and Sulindac was carried out using the human lung adenocarcinoma cell line Calu-3 and the human colon adenocarcinoma cell line Caco-2. Both cell lines express the ACE2 receptor and represent the anatomical sites (pulmonary and colon) of SARS-CoV-2 infection in humans [6,46,47]. To investigate the effect of SARS-CoV-2 infection on the host’s polyamine metabolism, we infected Calu-3 cells with the virus at 0.05 MOI and measured the mRNA levels of polyamine metabolic enzymes at 72 h post-infection. The mRNA level of a key polyamine biosynthetic enzyme, ODC1, was significantly elevated in the infected cells compared to uninfected (control) cells (more than a 9-fold increase, *p* = 0.03; Figure 1A, left panel). Similarly, the mRNA levels of the polyamine-catabolizing enzymes SAT1 and SMOX were elevated by 5-fold (*p* = 0.0007 and *p* = 0.008, respectively), which indicates that SARS-CoV-2 induces polyamine metabolism in infected cells. Treatment of infected Calu-3 cells with DFMO and Sulindac at the concentrations that suppress intracellular polyamine levels and inhibit proliferation or induce apoptosis in these cancer cells [28,48,49] significantly suppressed the RNA levels of the SAT1 and SMOX genes (*p* = 0.0007, *p* = 0.002, respectively), while the ODC1 mRNA level was not affected (Figure 1A, right panel).

When the infected Calu-2 cells were incubated in the presence of DFMO and/or Sulindac, the copy number of SARS-CoV-2 N1 RNA was significantly decreased in the conditioned media of the cell cultures treated with Sulindac alone and DFMO/Sulindac (on average, a 7-fold and a 10-fold decrease, respectively) (Figure 1B, left panel; *p* < 0.0001). Analysis of N1 copy number in the lysed cells showed a 2-fold reduction by the DFMO/Sul combination, but not the single agents (Figure 1B, right panel; *p* < 0.0001). A two-fold increase in the N1 transcript copy number was also noted in cell lysates when cells were treated with DFMO alone compared to the untreated cells or cells treated with Sulindac or the DFMO/Sul combination (*p* < 0.0001). This finding suggests that DFMO treatment suppresses viral RNA processing, leading to the accumulation of viral RNA within infected cells.

SARS-CoV-2 infection relies on the interaction between the Spike protein and the host’s ACE2 receptor protein. We measured the level of SARS-CoV-2 Spike mRNA in cell lysates and found it to be reduced by DFMO treatment (5-fold reduction, *p* = 0.02) and Sulindac treatment (more than a 27-fold reduction, *p* < 0.001) (Figure 1C, left panel). The combination treatment resulted in a 15-fold decrease in Spike mRNA level in treated cells (Figure 1C, left panel; *p* < 0.001). Sulindac alone and the DFMO/Sulindac combination also significantly reduced *ACE2* mRNA levels in the infected Calu-3 cells (by 5-fold and 29-fold, respectively) (Figure 1C, right panel; *p* < 0.003).

We also noted that DFMO and Sulindac act differently on the polyamine metabolic enzymes in uninfected (control) and infected Calu-3 cells. Particularly, *ODC1* and *SMOX* mRNA levels were not significantly altered in the uninfected Calu-3 cells treated with DFMO and Sulindac as single agents, while the DFMO/Sulindac combination significantly increased the *ODC1* transcript level. The *SAT1* mRNA level was significantly decreased in uninfected DFMO-treated cells at the concentrations of 2.5 mM and 5 mM, as well as with the DFMO/Sul combination at 1 mM/100 μM and 2.5 mM/200 μM (Figure S2A, *p* < 0.02). The *ACE2* mRNA level was significantly suppressed in uninfected Calu-3 cells treated with various concentrations of DFMO and Sulindac (Figure S2B). Significant suppression of SARS-CoV-2 N1 and Spike viral gene expression was also observed in the infected Caco-2 colon adenocarcinoma cell line treated with the various concentrations of DFMO and Sulindac (*p* < 0.001, Figure S3A,B).

The evaluation of DFMO and Sulindac interaction in Calu-3, Caco-2 and Vero cell lines was performed as described in the Section 2 and the results showed that DFMO combined with Sulindac had the stronger antiviral activity against SARS-CoV-2 when the cells were pretreated with these drugs before being infected (produced synergistic antiviral effect), while an additive antiviral effect of the DFMO and Sulindac combination was observed after cells were infected (Supplementary Data and Figure S1A–C).

### 3.2. Evaluation of DFMO and Sulindac Prophylaxis in K18-hACE2 Mouse Model of COVID-19

**Figure 2:**
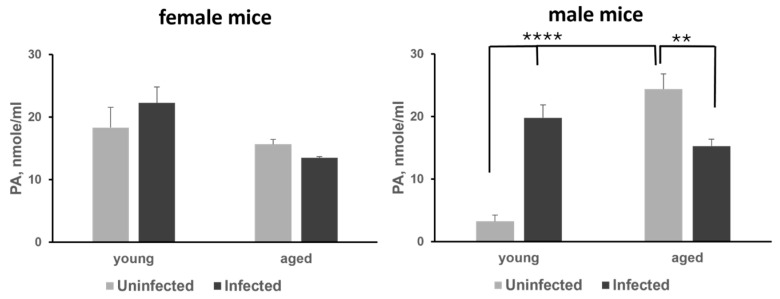
Analysis of the plasma polyamine (PA) levels in young and aged female (**left panel**) and male (**right panel**) mice. The *p*-values for uninfected vs. infected and young vs. aged groups of animals were determined by one-way ANOVA/two-sample *t*-test, and a *p*-value *p* < 0.05 was considered significant. ** *p* < 0.01, **** *p* < 0.0001.

We measured plasma polyamine levels in female and male *K18-hACE2* mice from two age groups: 6-week-old (young) and 58-week-old (aged), infected with SARS-CoV-2 at a dose of 1000 PFU on Day 7 post-infection. The polyamine levels in infected animals were compared to those in uninfected animals of the same age group (*n* = 4 animals per group). All animals were fed an AIN-93G diet. Analysis of plasma polyamine content in female mice showed no significant differences between uninfected and SARS-CoV-2-infected animals in either the young or aged groups (Figure 2, left panel, and Table S1A,B). In contrast, the infected 6-week-old male mice exhibited more than a 5-fold increase in plasma polyamine levels compared to uninfected males, mainly due to an increase in putrescine levels (*p* < 0.0001), which is synthesized from ornithine by ODC1 (Figure 2, right panel, and Table S1A). Additionally, we noted that plasma polyamine levels in uninfected aged male mice were significantly higher compared to uninfected young males. SARS-CoV-2 infection in aged mice resulted in decreased plasma polyamine levels, primarily due to a reduction in putrescine levels (*p* < 0.01) (Table S1B).

Because we observed sex and age differences in plasma polyamine levels in the infected *K18-hACE2* mice, we continued the analysis of DFMO and Sulindac antiviral efficacy within sex and age categories. The agents were tested as preventive measures (prophylaxis) and as post-infection treatment (treatment).

#### 3.2.1. Effect of DFMO and Sulindac Prophylaxis on the Survival Rates and Plasma Polyamine Levels in Young Animals

**Figure 3:**
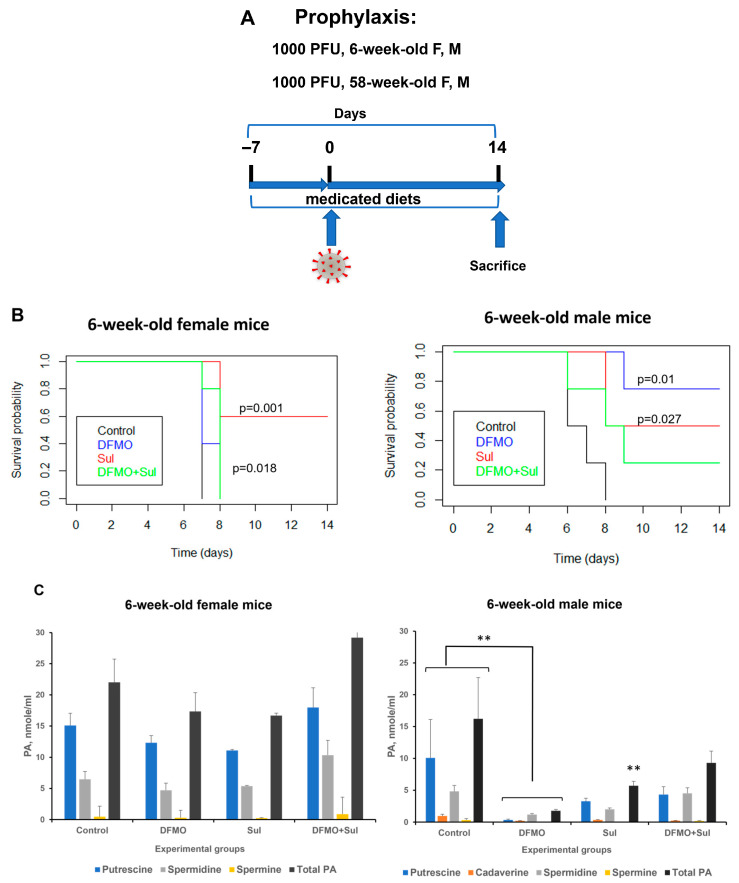
Effects of DFMO and Sulindac prophylaxis on survival and plasma polyamine levels in young (6-week-old) *K18-hACE2* mice. (**A**). Prophylaxis regimen scheme. Mice received AIN93G diet without drug supplementation (Control) or AIN93G diet supplemented with 835 ppm DFMO (DFMO), 167 ppm Sulindac (Sul), or 835 ppm DFMO and 167 ppm Sulindac (DFMO + Sul) for 7 days prior to infection with 1000 PFU of SARS-CoV-2. (**B**). Kaplan–Meier curves with *p*-values of survival rates for young female and male mice. The difference in survival rate was determined using a long-rank test and Cox proportion hazard test. (**C**). Polyamine (PA) levels in plasma of young female and male mice. The *p*-values for control vs. treatment groups were determined by one-way ANOVA/two-sample *t*-test, and a *p*-value *p* < 0.05 was considered significant. ** *p* < 0.01.

The 6-week-old *K18-hACE2* female and male mice (five animals per group) were placed on each of the four diets (AIN-93G, AIN-93G DFMO 835 ppm, AIN93G Sulindac 167 ppm, and AIN-93G DFMO/Sulindac combo) for 1 week before intranasal infection with 1000 PFU SARS-CoV-2, with continued drug supplementation as a medicated diet throughout the remainder of the experiment to a maximum of 14 days post-infection (dpi) (Figure 3A). 

*Survival analysis.* The survival rate of young untreated female mice was 0 at 14 dpi (mean survival of 7 dpi). The survival rates did not improve in young female mice receiving a DFMO alone diet (Figure 3B, 6-week female mice). The young female mice treated with Sulindac alone had a statistically significant increase in survival (mean survival of 11.6 dpi, *p* = 0.001; Table S2A). The young female mice treated with the DFMO/Sulindac combination also showed a statistically significant increase in survival compared to the untreated mice (mean survival of 7.8 dpi, *p* = 0.018). The Kaplan–Meier curve analysis in young male mice showed a statistically significant increase in the survival of the animals which received a DFMO-supplemented diet or Sulindac-supplemented diet but not in those which received a combination diet (Figure 3B, 6-week male mice). In particular, while untreated male mice developed morbidity symptoms by 8 dpi (mean survival of 6.75 dpi), the DFMO-treated group had a mean survival of 12.75 dpi (*p* = 0.01), and mice treated with Sulindac had a mean survival of 11.00 dpi (*p* = 0.027). The DFMO/Sulindac combination group had a mean survival of 9.25 dpi, although the difference in survival did not reach statistical significance (Table S2B, *p* = 0.077). The statistical analysis also revealed that all treatment groups had a hazard ratio of less than 1 compared to the infected untreated animals, and female mice fed a Sulindac-supplemented diet and male mice fed a DFMO-supplemented diet had the lowest hazard ratios when compared to other treatment groups (Table S2A,B). This finding indicates that the infected Sulindac-treated female animals and DFMO-treated male animals were less likely to die when compared to the untreated animals or animals in other treatment groups.

*Plasma polyamines analysis.* No statistically significant differences in plasma polyamine levels were found between untreated and treated groups in the young female mice (Figure 3C, 6-week-old female mice, Tables S3–S7). The statistical analysis of polyamine content in the plasma samples of young male mice identified a significant reduction in putrescine (*p* = 0.048, DFMO-treated group), cadaverine (*p* = 0.007 all treatment groups), spermine (*p* = 0.01, DFMO-treated group, and *p* = 0.038, Sulindac-treated group), and total polyamine content (*p* = 0.008, DFMO-treated group) compared to untreated mice (Figure 3C, 6-week-old male mice, and Tables S3–S7). The statistically significant sex–treatment interaction between untreated and treated 6-week-old male mice was found for plasma spermidine levels (Table S6).

#### 3.2.2. Effect of DFMO and Sulindac Prophylaxis on Survival Rates and Plasma Polyamine Levels in Aged Mice

**Figure 4:**
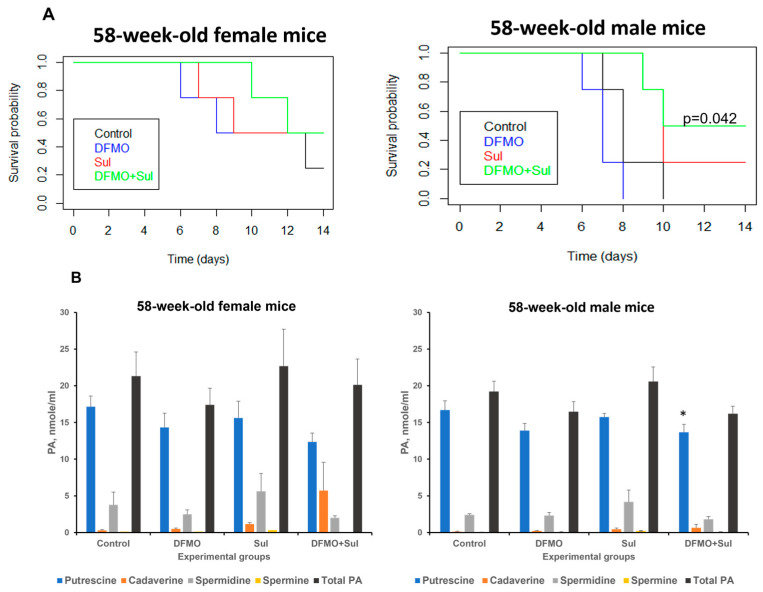
Effects of DFMO and Sulindac prophylaxis on survival and plasma polyamine levels in aged (58-week-old) *K18-hACE2* mice. Mice received AIN93G diet without drug supplementation (Control) or AIN93G diets supplemented with 835 ppm DFMO (DFMO), 167 ppm Sulindac (Sul), or 835 ppm DFMO and 167 ppm Sulindac (DFMO + Sul) for 7 days prior to infection with 1000 PFU of SARS-CoV-2. The medicated diets were provided throughout the duration of the experiments (14 dpi). (**A**). Kaplan–Meier curves with *p*-values of survival rates for aged female and male mice. The difference in survival rates was determined using the log-rank test and Cox proportion hazard test. A *p* ≤ 0.05 value between the control and treatment groups was considered significant. (**B**). Polyamine (PA) content in plasma of aged female and male mice. The *p*-values for control vs. treatment groups were determined by one-way ANOVA/two-sample *t*-test. * *p* = 0.053.

Similarly to the young mice described above, 58-week-old *K18-hACE2* mice were fed medicated diets for 7 days before intranasal infection with 1000 PFU SARS-CoV-2, with continued drug supplementation in the medicated diets throughout the experiment (14 dpi). Survival analysis showed that untreated aged female mice lived slightly longer than untreated aged male mice (the mean survival time for females was 10.5 dpi versus 8.25 dpi for males) (Figure 4, Table S8A,B). The DFMO/Sulindac-treated female mice had the longest average survival of 12 dpi (Figure 4A, left panel). The survival rate of untreated and DFMO-treated aged male mice was 0 at 14 days post-infection, whereas the Sulindac-treated and DFMO/Sulindac combination groups had survival rates of 0.25 and 0.50, respectively (Figure 4A, right panel). The shortest mean survival was observed in DFMO-treated male mice at 7 dpi, compared to other groups (Table S8B). Although Sulindac-treated aged male mice survived longer than untreated mice (mean: 10.75 dpi, *p* = 0.097), the only group with a statistically significant increase in survival was the DFMO/Sulindac group, with a mean survival of 11.75 dpi (*p* = 0.042, Table S8B). Hazard ratio analysis showed values less than 1 in all treatment groups except for DFMO-treated male mice. A decreasing trend in hazard ratio was noted in male mice; for example, male mice treated with Sulindac alone and DFMO/Sulindac had HRs of 0.27 and 0.16, respectively, indicating a significantly lower risk of death in these groups. Plasma polyamine analysis revealed that total plasma polyamine levels did not change significantly in infected animals with any treatment compared to untreated controls (Figure 4B, right panel, and Tables S9–S13). The marginally significant decrease was observed in plasma putrescine levels in the DFMO-treated (*p* = 0.063) and the DFMO/Sulindac-treated (*p* = 0.053) aged male mouse groups (Table S9).

#### 3.2.3. Effects of DFMO and Sulindac Prophylaxis on Animal Body Weights, Clinical Symptoms, and Lung Inflammation

We monitored the weight of the experimental animals which received the control or medicated AIN-93G diet before and after SARS-CoV-2 infection. There were no statistically significant differences in the weights of the animals in the different experimental groups on Day 0 (Tables S14–S17). The weight monitoring after infection showed that the weight loss in male mice was statistically significant on Day 7 post-infection (Figure S4A, *p* < 0.001). This decrease in weight was probably a result of an aversion to eating as clinical signs of infection worsened. Young female mice which received the Sulindac-containing diet and survived to Day 7 stabilized and regained their weights by the end of the experiment on Day 14 (Figure S4A, 6-week-old female mice). No statistically significant difference in mean daily weight change was found between the control and treatment groups in young female mice. A similar pattern of weight loss was observed in young male mice, with a statistically significant weight recovery in mice treated with DFMO, Sulindac alone, and the DFMO/Sulindac combination (*p* < 0.001) (Figure S4A, 6-week-old male mice). The analysis of mean daily weight changes in aged animals showed a similar pattern of weight loss, with the biggest decline by Day 9 in DFMO/Sulindac-treated mice of both sexes, following a significant regain of weight in mice that remained alive (Figure S4B). The weight changes were statistically significant in aged female mice, but not in male mice (*p* < 0.01; Figure S4B, 58-week-old female mice).

Daily weighing of the animals indicated that all infected animals lost weight during the first four days post-infection. Therefore, we compared the mean weight changes between the infected, untreated, and treated animals on Day 4 post-infection. We found significantly fewer changes in the weights of the young male mice receiving the DFMO/Sulindac-medicated diet compared to the DFMO-medicated diet (*p* = 0.03, Table S15).

The severity of clinical symptoms was assessed in the infected untreated (Control) and treated experimental groups using non-invasive clinical symptom scoring (Tables S18–S23). In young female mice, the clinical scores were significantly lower only in animals that received a Sulindac-containing diet (*p* = 0.003) (Table S18). In young male mice, the clinical scores were not significantly altered by any treatment (Table S19). In aged mice of both sexes, clinical scores did not improve significantly with any treatment (Tables S20 and S21). We also analyzed the interaction between treatment and sex in animals receiving prophylaxis regimen, and a trend toward a statistically significant effect was observed for young mice *(p*-value 0.081), while no interaction between treatment and sex was seen in aged mice (*p*-value 0.287). Overall, the changes in animal weights and clinical scores corresponded to the animal survival rates and improved in young female mice that received a Sulindac-containing diet.

During the histological examination of H&E-stained sections of lung tissue, no alveolar edema or airway necrosis was found in the infected, untreated (Control), or treated animals. The analysis of the lung tissue inflammation score determined a significant decrease in the level of alveolar inflammation in the lungs of the young Sulindac-treated animals of both sexes (*p* ≤ 0.01; Figure S5, 6-week-old mice) and in the level of peribronchiolar inflammation in young male mice fed a Sulindac-containing diet (*p* = 0.04). However, the total lung scores were not improved in young animals consuming medicated diets. The inflammation scores were not changed in the lung tissue of aged mice on a prophylaxis regimen (Figure S5, 58-week-old mice).

#### 3.2.4. Analysis of Sulindac and Sulindac Metabolite Contents in Plasma of Young and Aged Animals on Prophylaxis Regimen

The NSAID Sulindac is a prodrug, and it is metabolized in the liver into two forms: Sulindac sulfide, the non-selective inhibitor of COX-2, and Sulindac sulfone, which does not have anti-inflammatory activity but induces the polyamine catabolic enzyme SAT1 [28].

**Figure 5:**
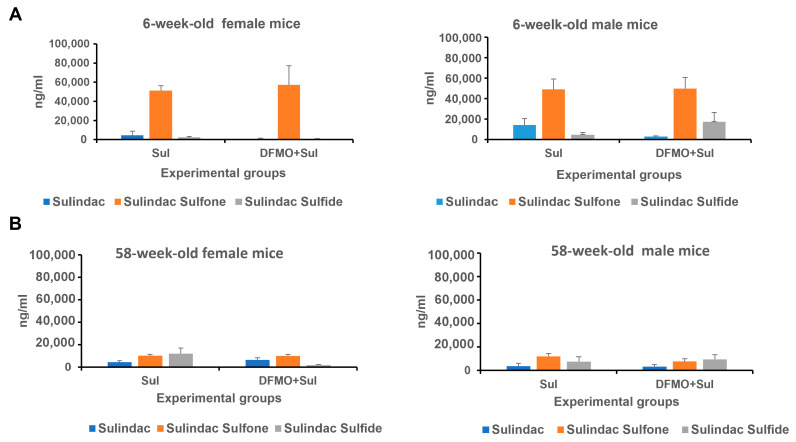
The levels of Sulindac and its metabolites Sulindac sulfone and Sulindac sulfide in young (6-week-old) (**A**) and aged (58-week-old) (**B**) *K18-hACE2* mice receiving prophylaxis regimen with Sulindac alone (Sul) or DFMO/Sul combo (DFMO + Sul). Data were analyzed using one-way ANOVA/two-sample *t*-test. No statistically significant difference was found between the Sulindac group and DFMO/Sul combo group within each sex.

Analysis of Sulindac and its metabolites in the plasma of mice treated with Sulindac alone or the DFMO/Sulindac combination is shown in Figure 5 and Tables S22–S27. The young mice of both sexes had similar levels of Sulindac sulfone in plasma, which were five-times higher than the Sulindac sulfone levels in aged mice (Figure 5A, Tables S22–S24). Among young animals, the Sulindac-treated mice exhibited higher plasma levels of Sulindac compared to the DFMO/Sulindac-treated group, although this difference did not reach statistical significance (*p* = 0.068). Six-week-old male mice treated with the DFMO/Sulindac combination had, on average, twice the amount of Sulindac and three times the amount of Sulindac sulfide in plasma compared to young females in the same group. The levels of Sulindac sulfone were similar between Sulindac-treated and DFMO/Sulindac-treated groups of both sexes. This suggests that the DFMO/Sulindac combination prophylaxis in young mice promotes a high rate of Sulindac oxidation into Sulindac sulfone in young males, but not in young females. Plasma levels of Sulindac and Sulindac sulfide were comparable in aged female and male mice treated with either Sulindac or the DFMO/Sulindac combination (Figure 5B, Tables S25–S27). However, aged females treated with DFMO/Sulindac had lower plasma Sulindac sulfide levels than females treated with Sulindac alone (*p* = 0.067) (Table S27). The levels of Sulindac and its metabolites were similar in aged males treated with either Sulindac or the DFMO/Sulindac combination. Additionally, aged mice of both sexes showed significantly lower Sulindac sulfone levels compared to young mice when treated with Sulindac alone (Table S23 vs. Table S26). These findings suggest sex- and age-specific activity of Sulindac-metabolizing enzymes, including Sulindac reductase and the microsomal cytochrome system, in the livers of infected animals.

#### 3.2.5. Effects of DFMO and Sulindac Prophylaxis on SARS-CoV-2 Infectivity and Viral Load

To evaluate the ability of DFMO and Sulindac to suppress SARS-CoV-2 infectivity, we performed plaque-forming assays using lung tissue samples from infected mice untreated or treated with DFMO, Sulindac, or the DFMO/Sulindac combination. The plaque-forming assay is a quantitative method for measuring infectious SARS-CoV-2 and is considered a gold-standard assay for measuring virions capable of replication [35,50]. The plaque-forming assay was performed in Vero cells infected with serial dilutions of the homogenized lung tissue samples. Plaques were counted after 72 h of incubation in a methylcellulose-containing (1% *w*/*v*) cell culture medium. The effects of drug treatments on SARS-CoV-2 viral load were measured by the N1 transcript copy number in the homogenized lung tissue of mice that received DFMO and Sulindac under prophylaxis and treatment regimes.

**Figure 6:**
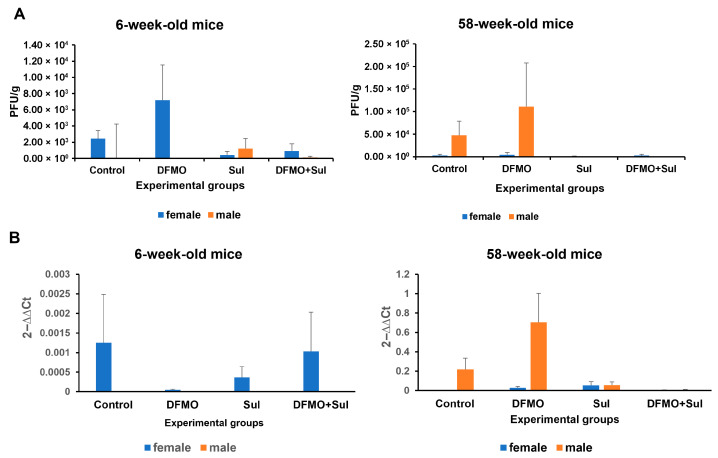
Effects of DFMO and Sulindac prophylaxis on SARS-CoV-2 infectivity and viral load in the lung tissue of infected *K18-hACE2* mice. Mice received an AIN93G diet without drug supplementation (Control) or AIN-93G diet supplemented with 835 ppm DFMO (DFMO), 167 ppm Sulindac (Sul), or 835 ppm DFMO and 167 ppm Sulindac (DFMO + Sul) for 7 days prior to infection with 1000 PFU of SARS-CoV-2. The medicated diets were provided throughout the experiments (14 dpi). (**A**). Plaque-forming assay results for the lung tissue of young (6-week-old mice) and aged (58-week-old) female and male mice. The results are shown as the number of plaque-forming units per g of lung tissue (PFU/g). (**B**). Analysis of SARS-CoV-2 N1 gene expression in the lung tissue of infected control and treated experimental groups. Data are presented as the normalized average N1 gene expression per group. Data were analyzed using an ANOVA single-factor test. No statistically significant difference was found between the control and treatment groups.

A high variability in the results of the plaque assay and N1 qPCR was noted among the infected, untreated, and treated mice which were placed on the prophylaxis regimen. Nevertheless, we found sex and age differences in infectivity and viral load among the experimental groups. In particular, the plaque count in the control and DFMO-treated groups was higher in the young female mice and aged male mice, while the mice treated with Sulindac alone and the DFMO/Sulindac combination developed fewer plaques, compared to the untreated groups (Figure 6A). The analysis of N1 transcript levels in the lung tissue of mice on the prophylaxis regimen showed more than a 25-fold decrease in young female mice treated with DFMO alone. The Sulindac alone and DFMO/Sulindac combination treatments did not have a significant effect on N1 transcript levels in these mice. The level of N1 transcripts in young male mice in all experimental groups was low (Figure 6B, 6-week-old mice). Moreover, no statistically significant difference was found between control and treatment groups. On the contrary, a difference in the N1 transcript level was noted between aged male and female mice, with 10-times higher N1 mRNA levels in male vs. female mice (Figure 6B, 58-week-old mice). The aged male mice treated with the DFMO/Sulindac combination had a more than 30-fold decrease in N1 transcript levels when compared to infected control male mice.

### 3.3. Evaluation of DFMO and Sulindac Treatment Regimens in K18-hACE2 Mouse Model of COVID-19

**Figure 7:**
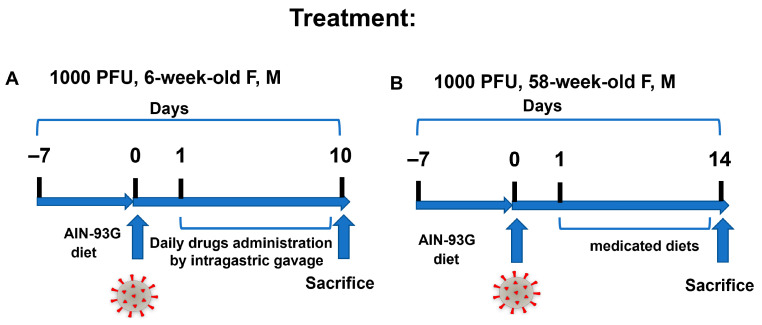
DFMO and Sulindac treatment regimens. (**A**). Treatment regimen scheme in young (6-week-old) *K18-hACE2* mice. The treatments started 24 h after animals were infected with 1000 PFU of SARS-CoV-2. The drugs were administered via daily intragastric gavage (IG) for the duration of the experiments (10 dpi). (**B**). Treatment regimen scheme for aged animals. Animals were switched to the control and medicated diets 24 h after being infected with 1000 PFU of SARS-CoV-2.

The *K18-hACE2* mice were maintained on an AIN-93G diet for 7 days before the intranasal infection with 1000 PFU of SARS-CoV-2. The DFMO and Sulindac administration started 24 h after the animals were infected with the virus. Young mice received drugs once daily by intragastric gavage (IG) and continued to a maximum of 10 dpi (Figure 7A). The selected treatment doses of 154.17 mg/kg (mg of compound per kg of body weight) of DFMO and 30.8 mg/kg of Sulindac were equivalent to the human doses tested in patients with prior colon polyps (ClinicalTrials.gov Identifier: NCT00118365) [30]. Control groups of young mice received the drug diluents (water control group) and 0.25 M sodium bicarbonate (sodium group). The infected aged mice were treated with the medicated diets (AIN-93G DFMO 835 ppm, AIN-93 G Sulindac 167 ppm, and AIN-93G DFMO/Sulindac combination) for the duration of the experiment (14 dpi) (Figure 7B).

*Survival Analysis*. The survival rates of young animals treated with Sulindac alone or in combination with DFMO were similar to those of the untreated (Control) mice, which indicates that no positive treatment effects were observed (Table S28). Similarly, none of the treated aged mice lived longer than the untreated mice (Table S29). The aged male mice showed a trend of increased survival with treatments compared to the untreated group, with survival rates of 0.5 in the DFMO-treated and Sulindac-treated groups and 0.75 in the DFMO/Sulindac group.

*Plasma polyamine analysis*. The plasma polyamine levels in young female mice did not significantly change with the treatments compared to the infected mock-treated group (administered water by IG) (Table S30). On the contrary, all infected 6-week-old male mice treated with a diluent (0.25 M sodium bicarbonate), DFMO, or Sulindac alone or in combination had significantly lower plasma levels of putrescine and total polyamines compared to the control mice (*p* < 0.0001, Table S31). The plasma polyamine contents were not significantly altered in 58-week-old infected female mice receiving the medicated diets compared to female mice on the control AIN-93G diet (Table S32). The 58-week-old male mice treated with Sulindac had the highest total polyamine levels in plasma (*p* < 0.01) compared to the untreated animals due to an increase in putrescine levels (*p* = 0.02) (Table S33).

No significant improvement in the clinical symptoms was found in the infected animals treated with DFMO or Sulindac (Tables S34–S37). The DFMO/Sulindac-treated aged female mice had the highest clinical score, and the DFMO/Sulindac-treated aged male mice had the lowest clinical score compared to other experimental groups (Tables S36 and S37). The average daily consumption of food (AIN93G diet control and medicated diets) gradually decreased in the infected animals in all experimental groups from 3.5 g/day (9.6% of body weight consumed) on the first day post-infection to 1.08 g/day (3.17% of body weight consumed) on Day 6 post-infection, and this decrease correlated with the increase in the clinical scores of the infected animals. Overall, the decrease in food consumption may explain the lack of improvement in infected mice receiving medicated diets. 

*Sulindac and Sulindac metabolite analysis*. We measured Sulindac and Sulindac metabolites in plasma samples of infected young mice treated with Sulindac or the DFMO/Sulindac combination and found no sex-related differences in metabolite levels between the treatment groups (Tables S38 and S39). The aged female mice had higher values of Sulindac sulfide and Sulindac sulfone in the Sulindac-treated group compared to the DFMO/Sulindac group, with Sulindac sulfone especially elevated in the Sulindac group (*p* = 0.09, Table S38). The aged animals had higher levels of Sulindac sulfone compared to the levels of Sulindac, which suggests that the administration of Sulindac via medicated diets resulted in its more efficient metabolic conversion in the liver, compared to its administration via intragastric gavage (Tables S40 and S41). 

*SARS-CoV-2 infectivity and viral load.* The results of the plaque-forming assays and the *N1* transcript levels in the lung tissue of young and aged mice on treatment regimens were inconclusive. We noted the significantly lower infectivity (10-fold lower) and the lower viral load (5-fold lower) in the lungs of the aged animals compared to the young ones. Additionally, the lung tissue samples from infected untreated mice exhibited a lower number of plaques and lower *N1* transcript levels when compared to the lung tissue of the treated animals (Figure S6A,B).

Overall, the tested treatment regimens did not significantly improve the disease outcome in any treatment groups, despite the improvement in the clinical scores of the aged male mice which received the DFMO and Sulindac combination treatment.

## 4. Discussion

Viruses rely on host metabolism to propagate; therefore, it is important to identify antiviral drugs that interfere with host cellular functions, which are required for the viral life cycle. There is solid experimental evidence that inhibition of the host’s polyamine biosynthesis can suppress the replication of the different viruses [9]. The novel coronavirus SARS-CoV-2 has unique genome differences compared to other coronaviruses; therefore, it was important to evaluate whether polyamine inhibition would be effective against this virus. DFMO is an irreversible inhibitor of the key polyamine biosynthetic enzyme ODC1. As an inhibitor of polyamine synthesis, DFMO has been widely used for the characterization of polyamine function in mammalian cells and has later been utilized as a clinical drug. DFMO is approved by the US Food and Drug Administration for the treatment of trypanosomiasis [51,52], hirsutism [53], and neuroblastoma [54]. Sulindac is an FDA-approved NSAID. Sulindac is converted by liver enzymes into Sulindac sulfide, which inhibits prostaglandin synthesis, and Sulindac sulfone, which does not have anti-inflammatory properties but induces polyamine catabolism [28]. The DFMO/Sulindac drug combination was successfully tested for its antitumor activity in an animal model of Familial Adenomatosis Polyposis [29]. The human clinical trial demonstrated that DFMO and Sulindac combination treatment is safe and highly effective in preventing the occurrence of colorectal adenomas in patients with prior colon polyps [30]. The drug combination can be safely administered as an oral medication in an outpatient setting at doses that reduce polyamine levels in adults as young as 18 years of age.

We assessed the susceptibility of SARS-CoV-2 to DFMO and Sulindac treatment to determine the potential of their use as an alternative approach to combat COVID-19. We tested the antiviral activity of these drugs against SARS-CoV-2 as single agents and in combination in human lung and colon cancer cell lines and in an animal model of COVID-19. In cell culture experiments, we showed that SARS-CoV-2 significantly induced the expression of key polyamine metabolic enzymes, *ODC1*, *SAT1*, and *SMOX*, in the infected cell lines. The expression of SARS-CoV-2 Spike and Nucleocapsid (N1) genes was significantly suppressed in cells treated with Sulindac alone or the DFMO/Sulindac combination (*p* < 0.0001). We determined that the DFMO/Sulindac drug combination synergistically suppressed SARS-CoV-2 viral load when cells were pretreated with the drugs for 48 h before exposure to the virus. The additive effect of the drug combination was observed when treatments were applied after infection. This finding indicates that polyamine depletion in cells prior to exposure to the virus improves the DFMO and Sulindac antiviral effect. We also found that Sulindac alone and the DFMO/Sulindac combination, but not DFMO alone, significantly reduced mRNA levels of the *ACE2* receptor.

The effectiveness of DFMO/Sulindac prophylaxis and treatment regimens against SARS-CoV-2 infection in vivo was studied using the *C57BL/6J K18-hACE2* transgenic mouse model. The analysis included the assessment of the clinical symptoms, body weight, and survival rates, as well as histopathology and biochemical endpoints specific to the tested drugs (plasma polyamine content and plasma Sulindac, Sulindac sulfone, and Sulindac sulfide contents). Measurement of SARS-CoV-2 viral load and plaque-forming ability in the lung tissue of the infected animals was performed to evaluate the effect of DFMO and Sulindac on SARS-CoV-2 replication. The *K18-hACE2* transgenic mice are highly susceptible to SARS-CoV-2 infection and accurately mimic COVID-19 symptoms in humans upon intranasal infection, including body weight loss, rapid breathing, fever, and inactivity [32] The infected mice typically reach criteria for euthanasia within 5–8 days post-infection when infected with SARS-CoV-2 at 1 × 10^5^ PFU [55]. We used an infection dose of 1000 PFU to observe the animals for a longer period post-infection (10–14 days). Analysis of plasma polyamine levels in *K18-hACE2* mice showed that plasma polyamine content was more significantly altered in infected male mice compared to female mice. The uninfected young male mice had lower plasma polyamine levels than the uninfected aged male and female mice. SARS-CoV-2 infection significantly increased polyamine contents in young infected male mice, mainly due to an increase in putrescine levels. The infected aged male mice had a significantly lower plasma putrescine level and lower total polyamine content than uninfected aged male mice. These sex-specific differences in polyamine levels in response to SARS-CoV-2 infection are important for evaluating the antiviral effectiveness of DFMO and Sulindac.

The prophylaxis regimen using drug supplementation through medicated diets resulted in notable sex- and age-specific differences in survival outcomes. Specifically, prophylaxis with DFMO and Sulindac as single agents was more effective in young male mice, significantly increasing their survival rates (*p* = 0.01, DFMO; *p* = 0.027, Sulindac). The combination of DFMO and Sulindac was marginally effective in young male mice (*p* = 0.077). Additionally, the DFMO/Sulindac combination prophylaxis, but not the individual drugs, significantly improved survival in aged male mice (*p* = 0.042). Young female mice benefited from the prophylaxis with Sulindac as a single agent, with an increase in survival rate from 0 to 0.6 (*p* = 0.001), and showed a statistically significant improvement in clinical scores (*p* = 0.003). The survival rates of the young female mice also significantly improved on the DFMO/Sulindac diet (*p* = 0.018). Low hazard ratios were observed in young male mice fed DFMO and young female mice fed Sulindac, indicating a shortened illness duration in these groups. These outcomes were supported by a significant recovery in body weight among infected animals treated with Sulindac alone. Lung histopathology revealed less alveolar and peribronchiolar inflammation in young animals on Sulindac prophylaxis, though the overall lung inflammation score did not significantly improve in aged animals. Plasma polyamine analysis of animals on the prophylaxis regimen showed that the DFMO diet significantly suppressed plasma polyamine levels in males (by eightfold compared to all other groups). A significant sex–treatment interaction was found for plasma spermine levels in 6-week-old male mice. In aged females, plasma polyamine levels were not significantly affected by medicated diets. Aged male mice on the DFMO/Sulindac combination diet, but not the other diets, showed a marginally significant decrease in plasma putrescine levels (*p* = 0.05).

The treatment regimen began with DFMO and Sulindac supplementation 24 h after infection, but it was less effective than prophylaxis. Notably, the survival rate analysis showed similar median survival times in young male and female mice. Although the combination of DFMO and Sulindac slightly increased the median survival times of aged male mice, overall survival in aged male and female mice did not significantly improve with any treatment. The regimen administered via intragastric gavage to infected young animals significantly reduced plasma polyamine levels in young male mice but not in young female mice. In contrast, aged animals receiving treatments through medicated diets showed no significant changes in plasma polyamine levels, except for aged males, who had higher polyamine contents on Sulindac-supplemented diets (*p* < 0.01). The lack of significant changes in plasma polyamine levels in animals on medicated diets may be due to decreased appetite and the development of anosmia after infection [55]. Overall, the low antiviral efficacy of DFMO and Sulindac in animals receiving the drugs as part of a treatment regimen could be a result of the delayed start of drug administration and also suggests that different dosing regimens should be tested.

The analysis of Sulindac metabolites in the plasma of animals which received prophylaxis with Sulindac alone or the DFMO/Sulindac combination showed that young animals of both sexes had, on average, five-times higher levels of Sulindac sulfone than aged animals. Additionally, prophylaxis with the DFMO/Sulindac combination resulted in a higher rate of Sulindac oxidation into Sulindac sulfide in both young and aged male mice, but not in female mice. These findings suggest possible sex- and age-specific activity of Sulindac-metabolizing enzymes, including Sulindac reductase and the microsomal cytochrome P450 system in the livers of infected animals [56]. Sulindac oxidation to Sulindac sulfone is catalyzed primarily by the microsomal cytochrome P450 system, and Sulindac sulfide is generated via reduction by the thioredoxin system. The observed differences in metabolite levels could be due to the different activities of these enzyme complexes in male and female mice, which can change with age. Sulindac sulfide metabolite inhibits the activities of the two isoforms (COX-1 and COX-2) of the cyclooxygenase enzyme, resulting in the suppression of prostaglandin levels [57], but they can also cause hepatic toxicity [58]. Sulindac can also induce apoptosis through both COX-dependent [59] and COX-independent mechanisms [28]. The sex- and age-dependent variations in the activity of Sulindac-metabolizing liver enzymes in some infected animals may cause a lower rate of production of Sulindac metabolites.

The analysis of the efficacy of DFMO and Sulindac against SARS-CoV-2 infectivity and viral load in animals on a prophylaxis regimen showed that animals which consumed the Sulindac only and the DFMO/Sulindac combination diets developed fewer plagues and had lower viral loads compared to untreated animals. This observation correlated with the improved survival in these experimental groups. By contrast, survival rates did not significantly improve in animals receiving the treatment regimens, probably due to the lack of response to the drugs at the tested concentrations and the high infectivity and high viral loads in the lung tissue samples of these animals.

The animal experiments also revealed variability in the animals’ responses to SARS-CoV-2 infection and the treatment regimens. Although we ensured the reproducibility of the animal studies by using a statistically justified number of animals, representing both sexes per treatment group and per time point, we observed variable viral loads among the infected mice and less than desired suppression of viral infection by DFMO/Sulindac in some experiments. This could be due to variability in the activity of the *K18* promoter, which controls the expression of hACE2 in target organs (lungs, brain, intestine, heart, liver, kidney, and spleen) of *K18-hACE2* transgenic animals. Additionally, the various degrees of anosmia in the SARS-CoV-2-infected animals [55] could have suppressed their appetite, resulting in a decrease in the consumption of medicated diets. The above factors contribute to the variability in treatment results in experimental animals.

Our experiments with the *K18-hACE2* transgenic mouse model of COVID-19 recapitulated the epidemiological reports regarding the higher vulnerability of men compared to women to COVID-19-related mortality. Both innate and adaptive immunity are regulated by sex hormones and affected by aging [60,61]. Therefore, sex hormones could contribute to the differences in animals’ responses to the treatments. Polyamines can alter the expression of pro-inflammatory genes and cytokine synthesis [62], as well as modulate an autoimmune response [63]. The modulation of the host’s immune response by polyamines during viral infection and by polyamine-targeted agents requires further investigation.

## 5. Conclusions

We found that DFMO and Sulindac significantly suppressed SARS-CoV-2 propagation and *ACE2* mRNA levels in the infected cell lines. The in vivo experiments revealed the sex- and age-dependent protective antiviral activity of DFMO and Sulindac when they were applied as part of a prophylaxis regimen. As prophylaxis, DFMO or Sulindac as single agents increased survival rates in the young male mice, and the DFMO/Sulindac combination was effective in the aged male mice. Young female mice demonstrated increased survival rates on the prophylaxis regimen with Sulindac alone and the DFMO/Sulindac combination, while aged female mice did not benefit significantly from any interventions. Interestingly, SARS-CoV-2-infected *K18 hACE2* mice exhibited both age- and sex-dependent differences when treated with the DFMO/Sulindac combo, but only sex-dependent differences were found in Sulindac metabolism.

The reduction in plasma polyamine levels in male mice on a prophylaxis regimen was associated with the suppression of the viral load and inflammation in the lung tissue. The prophylaxis with DFMO and Sulindac did not significantly alter plasma polyamine levels in female mice, but the DFMO- and Sulindac-fed groups had lower viral loads in the lungs compared to the untreated and DFMO/Sulindac-fed female mice.

This study demonstrated the positive results of DFMO and Sulindac prophylaxis of viral infection with specific protective age- and sex-dependent antiviral effects. The findings support further evaluation of the administration of DFMO and Sulindac as a preventive approach against SARS-CoV-2 infection for the population with vaccine hesitancy or upon development of resistance to the conventional antiviral treatments. Moreover, further efforts could be directed to repurposing DFMO and Sulindac as alternatives to antiviral medications for protection against long COVID symptoms.

## Data Availability

The original contributions presented in this study are included in the article/Supplementary Materials. Further inquiries can be directed to the corresponding author.

## Supplementary Materials

The following supporting information can be downloaded at https://www.mdpi.com/article/10.3390/v17101306/s1, Supplementary Materials: Figure S1. Results of SynergyFinder analysis of DFMO/Sulindac treatment in Calu-3 (A), Caco-2 (B), and Vero (C) cell lines infected with MOI 0.05. Figure S2. Gene expression analysis of uninfected Calu-3 lung adenocarcinoma cells treated with DFMO and Sulindac as single agents and in combination, measured by qPCR. Figure S3. Analysis of viral gene expression in Caco-2 colon adenocarcinoma cell line infected with SARS-CoV-2 virus at MOI 0.05 and treated with various concentrations of DFMO and Sulindac for 72 h. Figure S4. Effect of DFMO and Sulindac prophylaxis regimen on mean daily percent (%) weight change in young (6-week-old) and aged (58-week-old) *K18-hACE2* mice. Figure S5. Inflammation score analysis of the lung tissue of the infected animals on a prophylaxis regimen. Figure S6. Effects of DFMO and Sulindac treatment regimens on SARS-CoV-2 infectivity and viral load in the lung tissue of infected *K18-hACE2* mice. Tables. PROPHYLAXIS. Plasma polyamine analysis in control (uninfected) mice and mice infected with SARS-CoV-2 (MOI 1000 PFU) at 7 days post-infection (Table S1). PROPHYLAXIS. Summary of survival data for young mice on prophylaxis regimen (Table S2). Plasma polyamine analysis in young mice (Tables S3–S7). Summary of survival data for aged mice on prophylaxis regimen (Table S8). Plasma polyamine analysis in aged mice (Tables S9–S13). Daily weight change analysis (Tables S14–S17). Summary of clinical scores (Tables S18–S21). Sulindac and Sulindac metabolite analysis (Tables S22–S27). TREATMENT. Summary of survival data of young mice on treatment regimen (Table S28). Summary of survival data of aged mice on treatment regimen (Table S29). Plasma polyamine analysis in young and aged mice (Tables S30–S33). Summary of clinical scores (Tables S34–S37). Sulindac and Sulindac metabolite analysis in young (Tables S38 and S39) and aged mice (Tables S40 and S41).

## Abbreviations

The following abbreviations are used in this manuscript:

COVID-19	Coronavirus disease 2019
SARS-CoV-2	Severe acute respiratory syndrome coronavirus 2
GEM	Genetically engineered mouse
NSAID	Non-steroidal anti-inflammatory drug
DFMO	α-difluoromethylornithine
Sul	Sulindac
COX2	Cyclooxygenase 2
ODC1	Ornithine decarboxylase; SAT1, spermidine/spermine N1 acetyltransferase
SMOX	Spermine oxidase
N1	Nucleocapsid gene
DMEM	Dulbecco’s modified Eagle medium
MOI	Multiplicity of infection
PFU	Plaque-forming units
HPLC	High-performance liquid chromatography
