# Supplementary material for: Preclinical Evaluation of the Efficacy of α-Difluoromethylornithine and Sulindac Against SARS-CoV-2 Infection
Source: Viruses. 2025 Sep 26;17(10):1306. doi: 10.3390/v17101306 (PMC12567796; doi:10.3390/v17101306)
Supplement: Supplementary file 1 [file viruses-17-01306-s001.zip › viruses-3679616-supplementary.pdf]

viruses

MDPI

Supplementary Materials

1. Supplementary Data

Drug synergy analysis.

We assessed the drug synergy between DFMO and Sulindac in the Vero rhesus monkey kidney cell line, which is used for the propagation of SARS-CoV-2, and in the Calu-3 and Caco-2 human cell lines. The results of DFMO and Sulindac drug interaction analysis showed that combinations of DFMO/Sul act synergistically to suppress SARS-CoV-2 N1 transcript levels in in all three lines in a prophylaxis setting (Calu-3 cells Mean ZIP is 79,820,372.69, p=2.75e-1, Caco-2 cells Mean ZIP is 253.89, p=3.15e-02, and Vero cells Mean ZIP is 18.89, p=3.35e-02), and the drugs exert an additive inhibitory effect in a treatment setting (Calu-3 cells Mean ZIP score is -3.46, p=2.62e-01, Caco-2 cells Mean ZIP score is -6.68, p=3.75e-01, and Vero Mean ZIP score is -2.62, p=6.59e-01) (**Fig.S1A,B,C**).

2. Supplementary Figures

A

Calu-3

Prophylaxis

Drug	IC50, mM
DFMO	0.88
Sulindac	1.2

ZIP

Mean: 79820372.69 (p = 2.75e-01)

Treatment

Drug	IC50, mM
DFMO	2.06
Sulindac	0.86

ZIP

Mean: -3.46 (p = 2.62e-01)

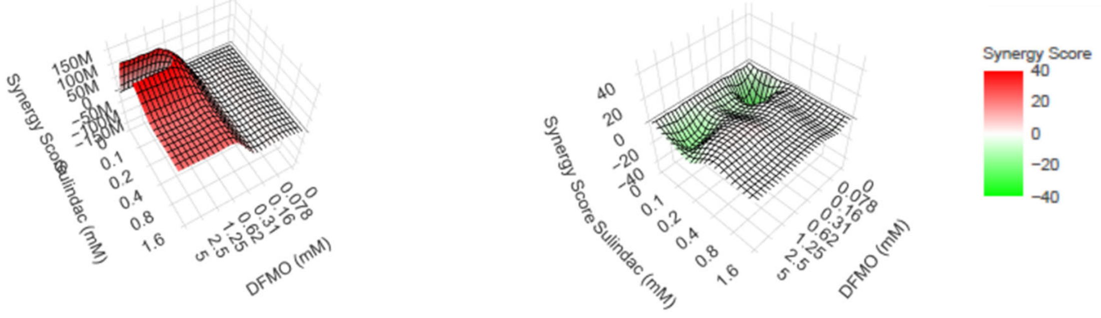

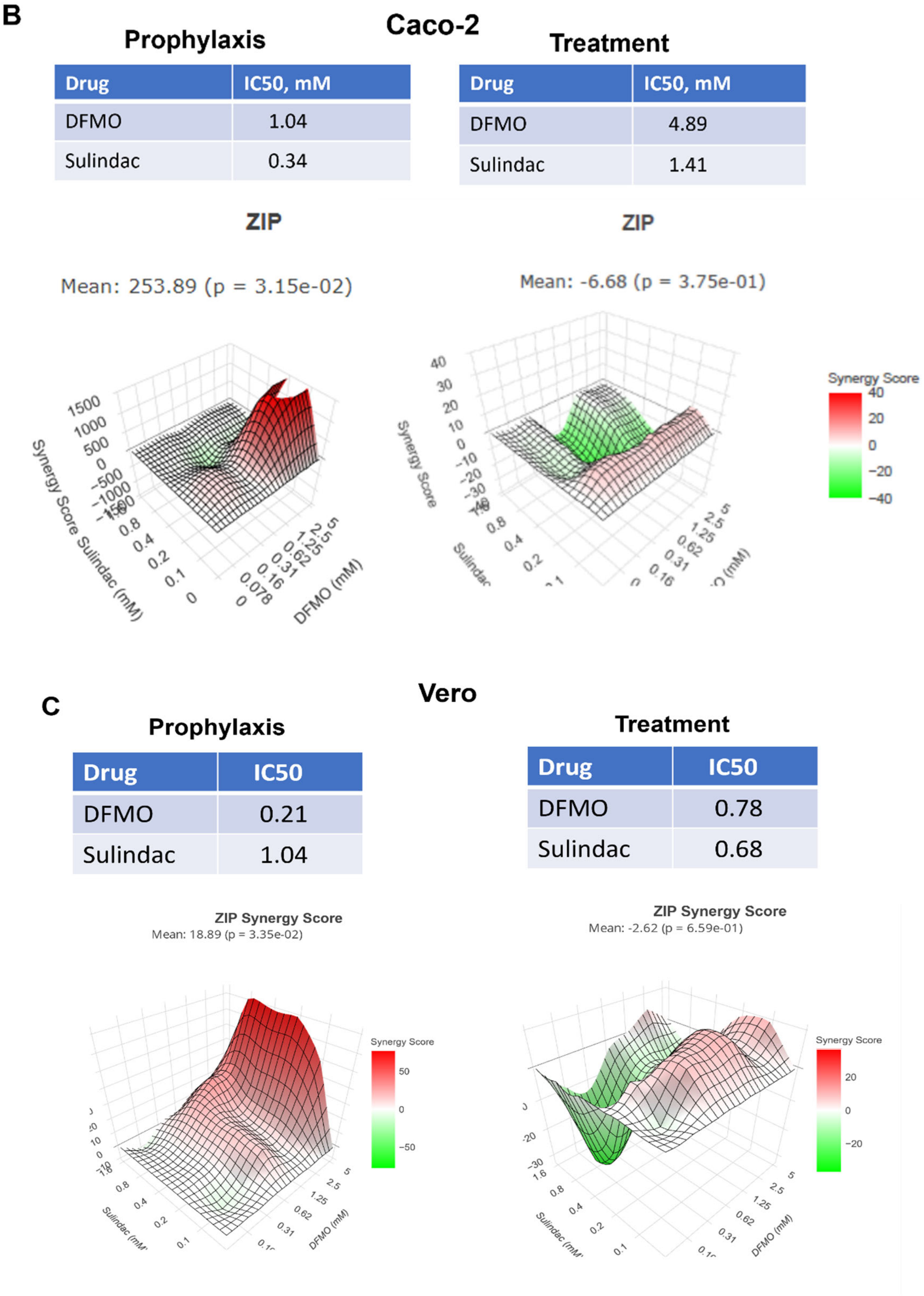

Figure S1. Results of SynergyFinder analysis of DFMO/Sulindac treatment in Calu-3 (A), Caco-2 (B), and Vero (C) cell lines, infected with MOI 0.05. Cells were incubated with different concentrations of DFMO and Sulindac for 48 hours before exposure to the virus (Prophylaxis) or DFMO and Sulindac were added to the cell culture media after cells were infected (Treatment). Cells were harvested at 72 hours post-infection, the virus was neutralized, and N1 transcript level was measured in the lysed cells by the qRT-PCR. DFMO and Sulindac interactions were analyzed using SynergyFinder web application as described in the Supplementary Methods section above. The half

maximum inhibitory concentrations (IC50) of DFMO and Sulindac in mM for each condition are shown. .

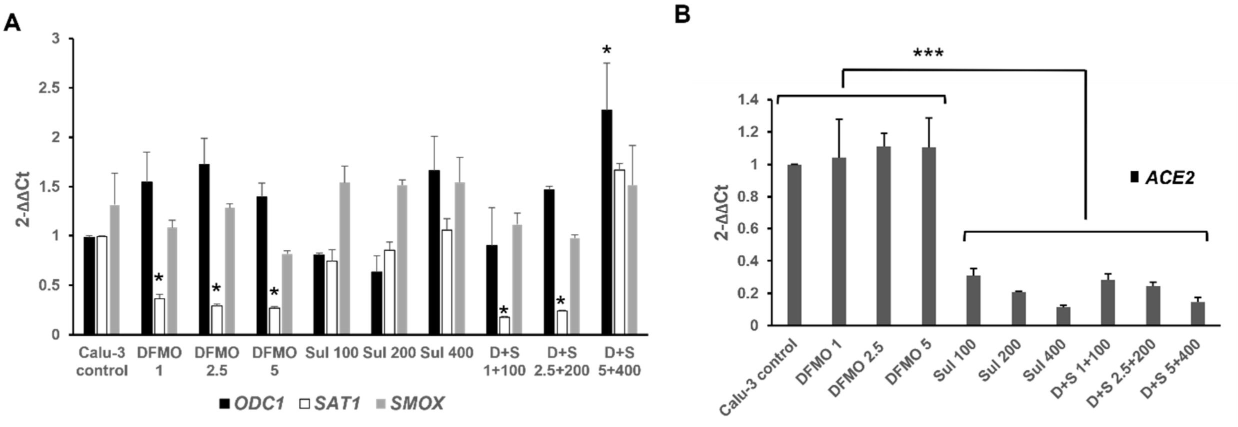

Figure S2. Gene expression analysis in uninfected Calu-3 lung adenocarcinoma cells treated with DFMO and Sulindac as single agents and in combination, measured by qPCR. **A.** Fold change in polyamine metabolic genes expression in Calu-3 cells incubated with the various concentrations of DFMO (1 mM, 2.5 mM, 5 mM), Sulindac (Sul) (100 μM, 200 μM, 400 μM), and DFMO/Sulindac combination (D+S) at 72 hours post-infection. **B.** Fold change in expression of *ACE2* mRNA in Calu-3 cells incubated with the various concentrations of DFMO (1 mM, 2.5 mM, 5 mM), Sulindac (Sul) (100 μM, 200 μM, 400 μM), and DFMO/Sulindac combination (D+S) for 72 hours. The data were analyzed using a single-factor ANOVA test. *p<0.02, ***p <0.001. Results representative of three independent experiments are shown.

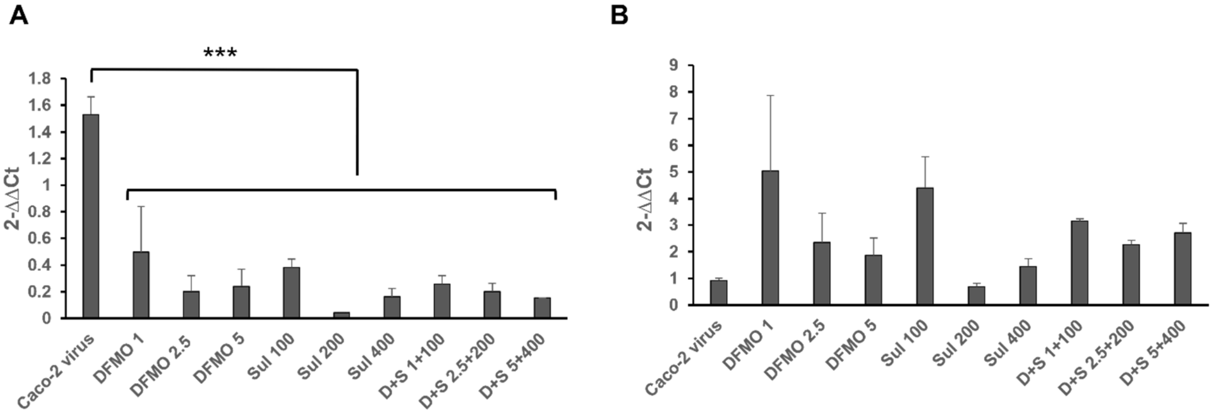

Figure S3. Analysis of viral gene expression in Caco-2 colon adenocarcinoma cell line infected with SARS-CoV-2 virus at MOI 0.05 and treated with various concentrations of DFMO (1 mM, 2.5 mM, 5 mM) and Sulindac (Sul) (100 μM, 200 μM, 400 μM) for 72 hours. **A.** Fold change in N1 mRNA by qPCR. **B**. Fold change in Spike mRNA level by qPCR. Data was analyzed using the ANOVA single-factor test. ***p<0.001.

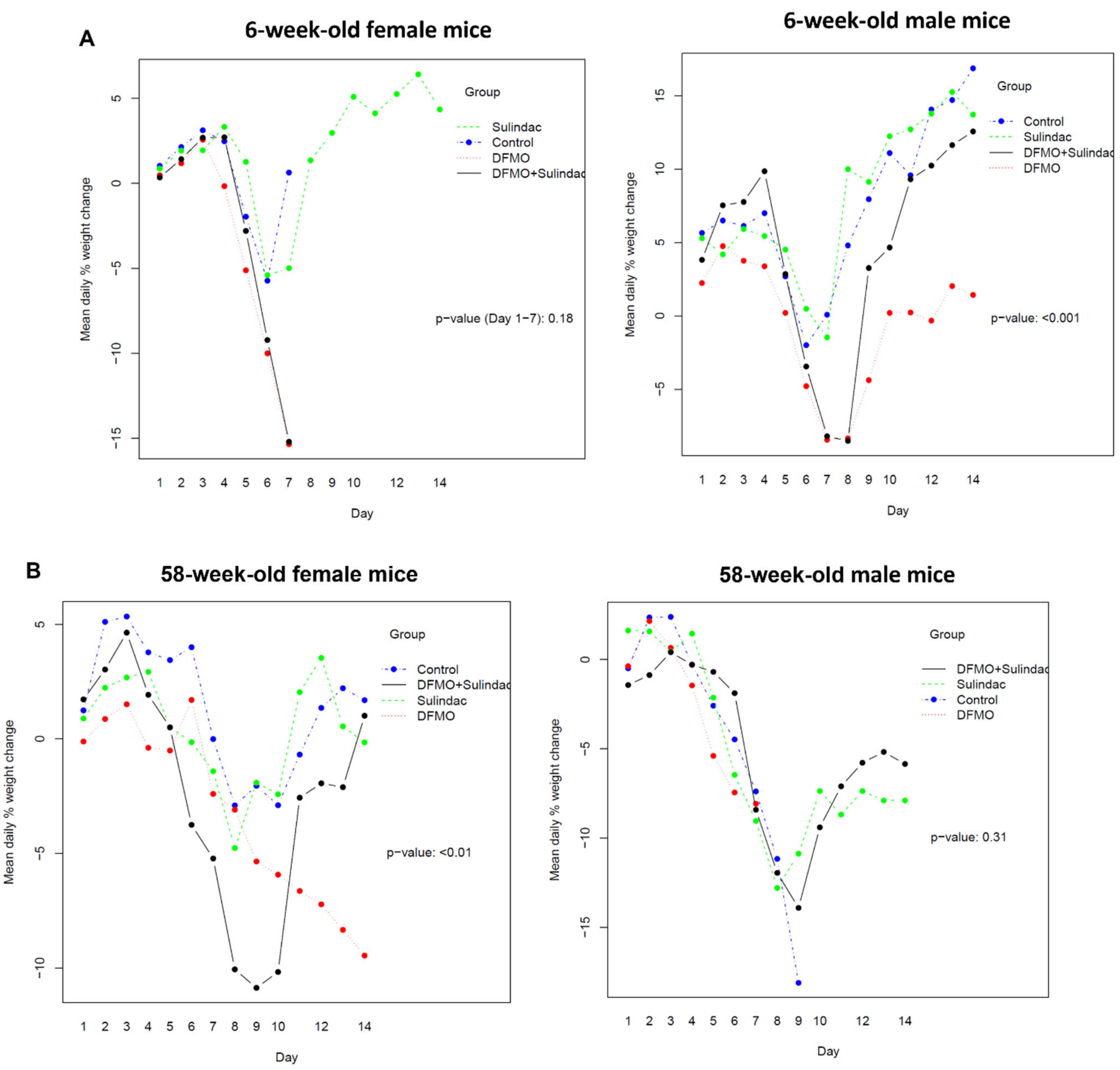

Figure S4. Effect of DFMO and Sulindac prophylaxis regimen on mean daily percent (%) weight change in young (6-week-old) and aged (58-week-old) *K18-hACE2* mice. Mice received AIN93G diet without drug supplementation (Control) or received the medicated diets with 835 ppm DFMO (DFMO), 167 ppm Sulindac (Sulindac), or 835 ppm DFMO and 167 ppm Sulindac (DFMO+Sulindac) for 7 days prior to infection with 1000 PFU. Mice's weights were measured daily during the experiment. Changes in daily mean percent weight change per group were analyzed using a linear mixed effects model with a random intercept. The p-values between the Control and treatment groups are presented for young and aged female and male mice.

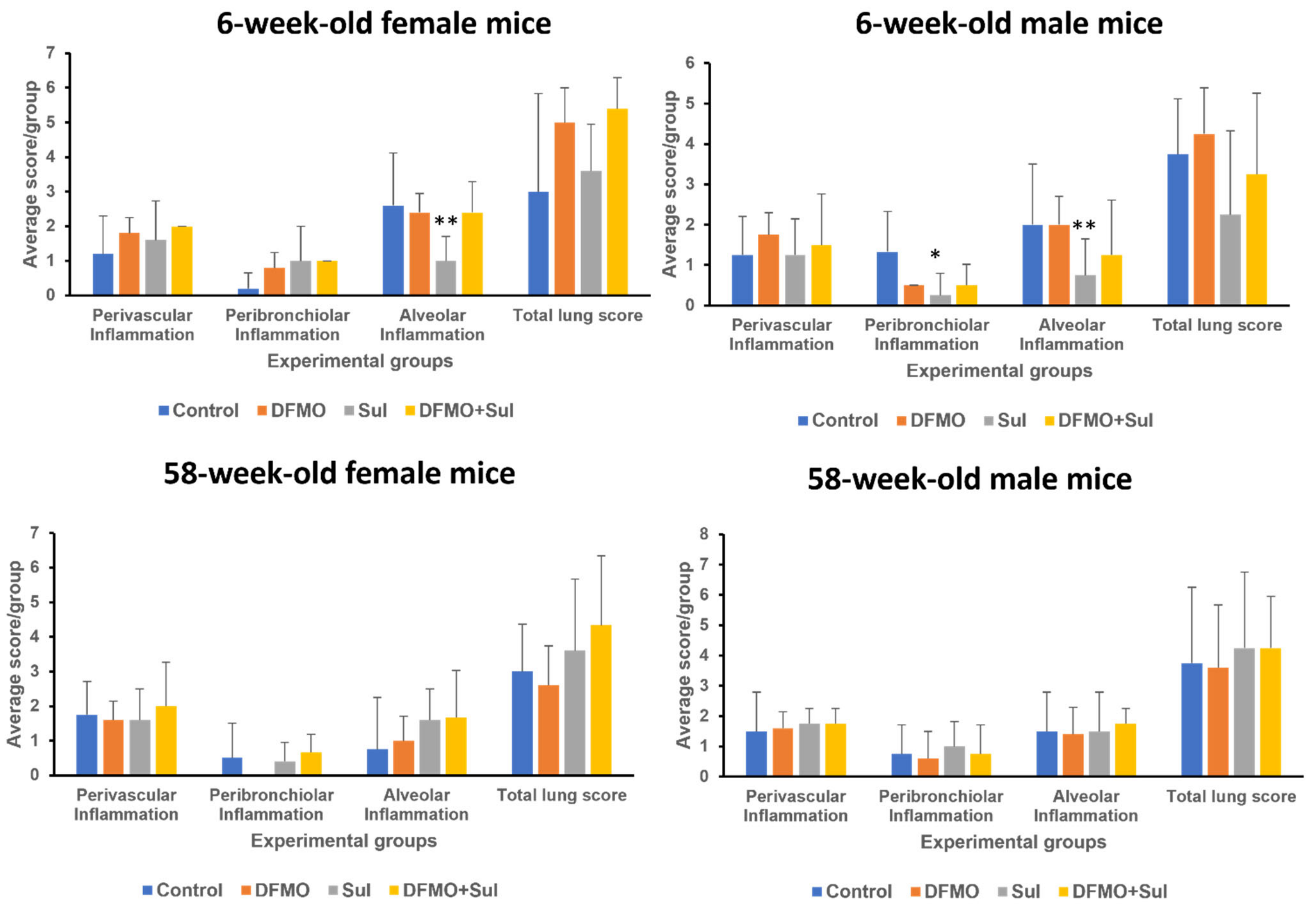

Figure S5. Inflammation score analysis in the lung tissue of the infected animals on a prophylaxis regimen. The inflammation scoring of the H&E-stained slides of lung tissue from young (6-week-old) and aged (58-week-old) female and male mice, untreated (Control) or receiving the DFMO, Sulindac (Sul), or DFMO/Sulindac combination (DFMO+Sul) diets, was performed as described in the Materials and Methods section. The data were analyzed using the ANOVA single-factor test. *p=0.04, **p ≤ 0.01.

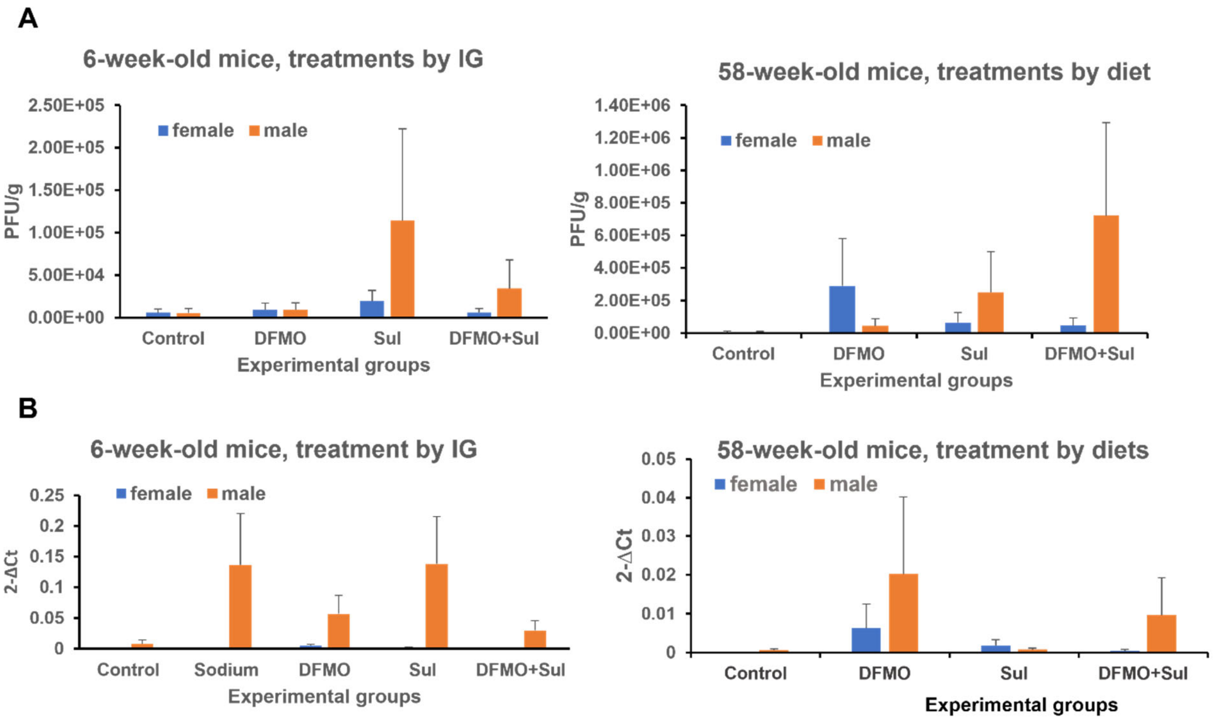

Figure S6. Effect of DFMO and Sulindac treatment regimens on SARS-CoV-2 infectivity and viral load in the lung tissue of infected *K18-hACE2* mice. **A**. Plaque-forming assay results in the lung tissue of the infected untreated (Control) and treated experimental groups of young (6-week-old mice) with the drug administration via intragastric gavage (IG) and aged (58-week-old) mice with the drug administration via medicated diets. The results are shown as the number of plaque-forming units per g of lung tissue (PFU/g). B. Analysis of SARS-CoV-2 N1 gene expression in the lung tissue of animals from the infected control and treated experimental groups. Data is presented as the normalized average N1 gene expression per group. Data was analyzed using ANOVA single-factor test. No statistically significant difference was found between the control and treatment groups.

3. Supplementary Tables.

Table S1. Polyamine content in the plasma of uninfected and infected with SARS-CoV-2 (MOI 1000 PFU) *K18-hACE2* mice by HPLC at 7 days post-infection. (4 animals/group, nmole/ml, Mean ±SE).

A. Polyamine content in the plasma of 6-week-old female and male *K18-hACE2* mice.

AIN-93G diet	Putrescine		Spermidine		Spermine		Total Polyamines	
	**female**	**male**	**female**	**male**	**female**	**male**	**female**	**male**
Uninfected	13.64±1.12	0.59±0.08	4.10±2.16	2.37±0.37	0.21±0.18	0.28±0.05	18.33±3.20	3.24±0.45
Infected, untreated	18.06±1.14	18.27±1.74****	3.78±1.42	1.47±0.31	0.21±0.17	0.03±0.01	22.26±2.56	19.78±2.05****

B. Polyamine content in the plasma of 58-week-old female and male *K18-hACE2* mice.

AIN-93G diet	Putrescine		Spermidine		Spermine		Total Polyamines	
	**female**	**male**	**female**	**male**	**female**	**male**	**female**	**male**
Uninfected	14.16±0.89	23.05±2.47	1.48±0.28	1.17±0.18	n.d.	0.14±0.07	15.64±0.77	24.37±2.43
Infected, untreated	12.05±0.36	14.11±1.04**	1.17±0.18	1.11±0.04	n.d.	0.04±0.02	13.47±0.23	15.26±1.10**

****p- value <0.0001; **p-value <0.01; n.d.-not detected

3.1. Prophylaxis

Table S2. Summary of survival data for young mice (control vs medicated diets) on prophylaxis regimen.

A. 6-week-old female mice.

Group	N	Survival[a]	Median (days)[b]	Mean (days)[c]	HR (vs. Control)[d] (95% CI)	p-value
Control	5	0.0	7	7.0		
DFMO	5	0.0	7	7.4	0.27 (0.062-1.172)	0.08
Sul	5	0.6	NA	11.6	0.03 (0.004-0.23)	0.001
DFMO+Sul	5	0.0	8	7.8	0.16 (0.033-0.726)	0.018

B. 6-week-old male mice.

Group	N	Survival[a]	Median (days)[b]	Mean (days)[c]	HR (vs. Control)[d] (95% CI)	p-value
Control	4	0.00	6.5	6.75		
DFMO	4	0.75	NA	12.75	0.04 (0.004-0.476)	0.01
Sul	4	0.50	8.0	11.00	0.12 (0.019-0.789)	0.027
DFMO+Sul	4	0.25	8.5	9.25	0.22 (0.041-1.175)	0.077

[a] survival rate at 14 days
[b] median survival time derived from Kaplan-Meier curves
[c] mean survival time derived from Kaplan-Meier curves
[d] hazard ratio (HR) derived from Cox regression

3.2. Plasma Polyamine Analysis in young mice (Tables S3-S7)

Table S3. Summary of Putrescine by treatment and sex for 6-week-old mice.

Group	Female)			p-value	Male			p-value	Interaction p-value
	N	Mean	SD		N	Mean	SD		
Control	5	15.08	4.36		3	3.42	2.20		0.525
DFMO	5	12.32	2.84	0.418	4	0.35	0.29	0.048	
Sul	5	11.08	3.76	0.247	4	3.28	1.08	0.924	
DFMO+Sul	5	17.97	8.34	0.398	4	4.34	2.74	0.518	

Table S4. Summary of Cadaverine by treatment for 6-week-old male mice.

Group	N(Male)	Mean(Male)	SD(Male)	p-
Control	3	0.69	0.39	
DFMO	4	0.21	0.09	0.007
Sul	4	0.36	0.05	0.045
DFMO+Sul	4	0.23	0.14	0.008

Table S5. Summary of Spermidine by treatment and sex for 6 week-old mice.

Group	Female			p-value	Male			p-value	Interaction p-value
	N	Mean	SD		N	Mean	SD		
Control	5	6.47	2.58		3	0.41	0.64		0.274
DFMO	5	4.73	2.53	0.507	4	0.04	0.02	0.112	
Sul	5	5.37	2.71	0.674	4	0.04	0.02	0.114	
DFMO+Sul	5	10.34	6.75	0.152	4	0.19	0.14	0.319	

Table S6. Summary of Spermine by treatment and sex for 6 week-old mice.

Group	Female			p-value	Male			p-value	Interaction p-value
	N	Mean	SD		N	Mean	SD		
Control	5	0.47	0.34		3	4.68	2.50		0.008
DFMO	5	0.29	0.27	0.564	4	1.20	0.34	0.010	
Sul	5	0.24	0.16	0.461	4	2.02	0.43	0.038	
DFMO+Sul	5	0.88	0.88	0.212	4	4.54	1.88	0.905	

Table S7. Summary of total polyamines by treatment and sex for 6-week-old mice.

Group	Female			p-value	Male			p-value	Interaction p-value
	N	Mean	SD		N	Mean	SD		
Control	5	22.03	7.04		3	9.19	4.29		0.558
DFMO	5	17.33	5.34	0.397	4	1.80	0.47	0.008	
Sul	5	16.69	6.06	0.337	4	5.71	1.54	0.152	
DFMO+Sul	5	29.19	13.28	0.203	4	9.29	4.17	0.968	

Note: p-value (vs. Control) by Sex was derived from one-way ANOVA and the interaction p-value between Treatment and Sex was derived from two-way ANOVA with the interaction terms between Treatment and Sex indicators

Table S8. Summary of survival data for aged mice (control vs medicated diets) on prophylaxis regimen. .

A. 58-week-old female mice.

Group	N	Survival[a]	Median (days)[b]	Mean (days)[c]	HR (vs. Control)[d] (95% CI)	p-value
Control	4	0.25	10.5	10.5		
DFMO	4	0.50	8.0	10.5	0.70 (0.117-4.199)	0.697
Sul	4	0.50	9.0	11.0	0.63 (0.104-3.748)	0.607
DFMO+Sul	4	0.50	12.0	12.5	0.49 (0.081-2.951)	0.435

B. 58-week-old male mice.

Group	N	Survival[a]	Median (days)[b]	Mean (days)[c]	HR (vs. Control)[d] (95% CI)	p-value
Control	4	0.00	8	8.25		
DFMO	4	0.00	7	7.00	4.70 (0.903-24.501)	0.066
Sul	4	0.25	10	10.75	0.27 (0.059-1.263)	0.097
DFMO+Sul	4	0.50	10	11.75	0.16 (0.029-0.937)	0.042

[a] survival rate at 14 days
[b] median survival time derived from Kaplan-Meier curves
[c] mean survival time derived from Kaplan-Meier curves
[d] hazard ratio derived from Cox regression

3.3. Plasma Polyamine Analysis in aged mice by treatment and sex (Tables S9-S13)

Table S9. Summary of Putrescine for 58 week-old mice.

Group	Female			p-value	Male			p-value	Interaction p-value
	N	Mean	SD		N	Mean	SD		
Control	4	17.16	2.90		4	16.69	2.51		0.804
DFMO	6	15.65	4.70	0.593	5	13.90	2.16	0.063	
Sul	6	15.62	5.59	0.586	4	15.73	1.02	0.520	
DFMO+Sul	6	12.37	2.91	0.102	4	13.68	2.15	0.053	

Table S10. Summary of Cadaverine for 58-week-old mice.

Group	Female			p-value	Male			p-value	Interaction p-value
	N	Mean	SD		N	Mean	SD		
Control	4	0.28	0.20		4	0.12	0.10		0.443
DFMO	6	0.48	0.33	0.952	5	0.22	0.14	0.772	
Sul	6	1.14	0.48	0.791	4	0.45	0.31	0.361	
DFMO+Sul	6	5.72	9.42	0.107	4	0.65	0.95	0.146	

Table S11. Summary of Spermidine for 58 week-old mice.

Group	Female			p-value	Male			p-value	Interaction p-value
	N	Mean	SD		N	Mean	SD		
Control	4	3.77	3.49		4	2.40	0.32		0.946
DFMO	6	2.59	1.48	0.613	5	2.30	0.98	0.929	
Sul	6	5.61	6.01	0.436	4	4.18	3.19	0.156	
DFMO+Sul	6	2.02	0.66	0.457	4	1.81	0.70	0.626	

Table S12. Summary of Spermine by treatment and sex for 58 week-old mice.

Group	Female			p-value	Male			p-value	Interaction p-value
	N	Mean	SD		N	Mean	SD		
Control	4	0.11	0.19		4	0.00	0.00		0.962
DFMO	6	0.10	0.12	0.973	5	0.07	0.10	0.669	
Sul	6	0.32	0.72	0.423	4	0.23	0.43	0.168	
DFMO+Sul	6	0.02	0.03	0.711	4	0.06	0.11	0.725	

Table S13. Summary of Total PA by treatment and sex for 58 week-old mice.

Group	Female			p-value	Male			p-value	Interaction p-value
	N	Mean	SD		N	Mean	SD		
Control	4	21.33	6.59		4	19.22	2.84		0.99
DFMO	6	18.82	5.55	0.667	5	16.49	3.08	0.207	
Sul	6	22.69	12.33	0.815	4	20.59	3.99	0.537	
DFMO+Sul	6	20.13	8.68	0.836	4	16.20	2.02	0.188	

Note: p-value (vs. Control by Sex was derived from one-way ANOVA and the interaction p-value between Treatment and Sex was derived from two-way ANOVA with the interaction terms between Treatment and Sex indicators

3.4. Daily Weight change analysis (Tables S14-S17)

Table S14. Summary of daily weight for 6-week-old female mice.

Group	N	Mean (D0)	SD (D0)	Mean (D4)	SD (D4)	Mean (% wt change)	SD (% wt change)
Control	5	16.28	1.05	16.70	1.47	2.48	3.75
DFMO	5	17.28	0.93	17.26	1.34	-0.16	4.57
Sulindac	5	17.56	0.87	18.14	0.90	3.33	2.37
DFMO+Sulindac	5	17.44	0.80	17.90	0.60	2.71	3.10
p-value (ANOVA)						0.47	

Table S15. Summary of daily weight for 6-week-old male mice.

Group	N	Mean (D0)	SD (D0)	Mean (D4)	SD (D4)	Mean (% wt change)	SD (% wt change)
Control	4	21.55	1.77	23.05	1.87	6.99	3.03
DFMO	4	22.25	1.58	23.02	2.08	3.37	2.29
Sulindac	4	23.30	1.22	24.58	1.64	5.43	2.75
DFMO+Sulindac	4	21.65	0.44	23.77	0.19	9.85	2.14
p-value (ANOVA)						0.03*	

*DFMO vs DFMO+Sulindac

Table S16. Summary of daily weight for 58-week-old female mice.

Group	N	Mean (D0)	SD (D0)	Mean (D4)	SD (D4)	Mean (% wt change)	SD (% wt change)
Control	4	30.40	4.06	31.38	2.50	3.78	6.34
DFMO	6	33.32	6.63	33.15	6.37	-0.39	1.67
Sulindac	6	30.02	4.46	30.83	4.09	2.93	2.21
DFMO+Sulindac	6	26.25	2.07	26.73	1.81	1.92	1.88
p-value (ANOVA)						0.11	

Table S17. Summary of daily weight for 58-week-old male mice.

Group	N	Mean (D0)	SD (D0)	Mean (D4)	SD (D4)	Mean (% wt change)	SD (% wt change)
Control	4	38.83	4.83	38.75	5.29	-0.28	2.73
DFMO	5	37.32	4.95	36.78	5.18	-1.45	4.46
Sulindac	4	37.15	4.14	37.67	4.02	1.46	0.88
DFMO+Sulindac	4	36.48	3.23	36.35	2.98	-0.29	1.72
p-value (ANOVA)						0.59	

3.5. Summary of clinical scores (Tables S18-S21)

Table S18. Summary of clinical score for 6-week-old female mice (untreated control vs medicated diets).

Group	N	Mean	SD	p-value (vs. Control)[a]
Control	5	8.2	1.30	
DFMO	5	9.0	1.41	0.581
Sul	5	3.2	3.96	0.003
DFMO+Sul	5	8.6	0.89	0.782

[a] derived from two-sample t-test

Table S19. Summary of clinical score for 6 week-old male mice (untreated control vs medicated diets).

Group	N	Mean	SD	p-value (vs. Control)[a]
Control	4	7.75	0.96	
DFMO	4	5.00	2.71	0.171
Sul	4	7.00	3.16	0.698
DFMO+Sul	4	7.75	3.20	1

[a] derived from two-sample t-test

Table S20. Summary of clinical score for 58 week-old female mice (untreated control vs medicated diets).

Group	N	Mean	SD	p-value (vs. Control)[a]
Control	4	6.25	3.59	
DFMO	4	6.00	2.45	0.92
Sul	4	6.25	4.43	1
DFMO+Sul	4	5.25	2.99	0.688

[a] derived from two-sample t-test

Table S21. Summary of clinical score for 58 week-old male mice (untreated control vs medicated diets).

Group	N	Mean	SD	p-value (vs. Control)[a]
Control	4	9.00	0.82	
DFMO	4	9.00	0.82	1
Sul	4	7.75	3.40	0.51
DFMO+Sul	4	6.25	3.78	0.161

[a] derived from two-sample t-test

3.6. Sulindac and Sulindac metabolites analysis in young and aged mice (Tables S22-S27)

Table S22. Summary of Sulindac by treatment and sex for 6-week-old mice.

Group	Female			p-value	Male			p-value	Interaction p-value
	N	Mean	SD		N	Mean	SD		
Sul	5	4422.61	3834.84		4	14069.74	12920.02		0.231
DFMO+Sul	5	771.212	472.31	0.068	4	2860.408	1459.87	0.135	

Table S23. Summary of Sulindac Sulfone by treatment and sex for 6-week-old mice.

Group	Female			p-value	Male			p-value	Interaction p-value
	N	Mean	SD		N	Mean	SD		
Sul	5	51319.37	11459.58		4	49018.98	20427.87		0.856
DFMO+Sul	5	57116.29	45160.93	0.788	4	49830.46	21678.88	0.958	

Table S24. Summary of Sulindac Sulfide by treatment and sex for 6-week-old mice.

Group	Female			p-value	Male			p-value	Interaction p-value
	N	Mean	SD		N	Mean	SD		
Sul	5	2346.27	1875.39		4	4760.514	4483.8		0.106
DFMO+Sul	5	671.91	532.70	0.091	4	17385.09	18187.49	0.226	

Note: p-value (vs. Sulindac) by Sex was derived from one-way ANOVA and the interaction p-value between Treatment and Sex was derived from two-way ANOVA with the interaction terms between Treatment and Sex indicators

Table S25. Summary of Sulindac by treatment and sex for 58-week-old mice.

Group	Female			p-value	Male			p-value	Interaction p-value
	N	Mean	SD		N	Main	SD		
Sul	6	4309.53	3411.94		4	3592.79	5075.91		0.523

Group	Female			p-value	Male			p-value	Interaction p-value
	N	Mean	SD		N	Main	SD		
DFMO+Sul	6	6473.44	4608.59	0.377	4	3247.38	3748.40	0.916	

Table S26. Summary of Sulindac Sulfone by treatment and sex for 58-week-old mice.

Group	Female			p-value	Male			p-value	Interaction p-value
	N	Mean	SD		N	Mean	SD		
Sul	6	10251.33	3089.00		4	11990.54	4782.92		0.258
DFMO+Sul	6	9975.72	3157.35	0.882	4	7649.15	4628.41	0.24	

Table S27. Summary of Sulindac Sulfide by treatment and sex for 58-week-old mice.

Group	Female			p-value	Male			p-value	Interaction p-value
	N	Mean	SD		N	Mean	SD		
Sul	6	11991.42	12108.76		4	7519.72	8306.09		0.135
DFMO+Sul	6	1792.62	1259.79	0.067	4	9432.33	7966.78	0.751	

Note: p-value (vs. Sulindac) by Sex was derived from one-way ANOVA and the interaction p-value between Treatment and Sex was derived from two-way ANOVA with the interaction terms between Treatment and Sex indicators

3.8. Treatment.

Table 28. Summary of survival data of young mice (control vs drugs by gavage) on treatment regimen.

A. 6-week-old female mice.

Group	N	Survival[a]	Median (days)[b]	Mean (days)[c]	HR (vs. Control)[d] (95% CI)	p-value
Control	3	0.0	7	6.67		
Sodium	5	0.6	NA	8.60	0.28 (0.05-1.68)	0.16
Sul	5	0.2	7	7.20	0.75 (0.17-3.39)	0.71
DFMO	5	0.2	6	6.80	1.09 (0.24-4.94)	0.91
DFMO+Sul	5	0.0	6	6.00	1.73 (0.4-7.36)	0.46

B. 6-week-old male mice.

Group	N	Survival[a]	Median (days)[b]	Mean (days)[c]	HR (vs. Control)[d] (95% CI)	p-value
Control	3	0.0	7	6.67		
Sodium	5	0.4	8	8.00	0.27 (0.05-1.47)	0.13
Sul	5	0.0	6	5.80	3.88 (0.76-19.89)	0.10
DFMO	5	0.4	7	7.80	0.33 (0.06-1.71)	0.19
DFMO+Sul	5	0.0	7	6.60	0.91 (0.22-3.82)	0.90

[a] survival rate at 10 days
[b] median survival time derived from Kaplan-Meier curves
[c] mean survival time derived from Kaplan-Meier curves
[d] hazard ratio (HR) derived from Cox regression

Table 29. Summary of survival data of aged mice (control vs medicated diets) on treatment regimen.

A. 58-week-old female mice.

Group	N	Survival[a]	Median (days)[b]	Mean (days)[c]	HR (vs. Control)[d] (95% CI)	p-value
Control	4	0.5	11	11.50		
DFMO	5	0.6	NA	11.60	0.902 (0.127-6.409)	0.918
Sul	5	0.4	10	11.00	1.344 (0.223-8.112)	0.747
DFMO+Sul	5	0.0	9	9.00	4.125 (0.718-23.706)	0.112

B. 58-week-old male mice.

Group	N	Survival[a]	Median (days)[b]	Mean (days)[c]	HR (vs. Control)[d] (95% CI)	p-value
Control	4	0.25	9	9.75		
DFMO	4	0.50	9	11.00	0.593 (0.099-3.559)	0.567
Sul	4	0.50	9	11.00	0.593 (0.099-3.559)	0.567
DFMO+Sul	4	0.75	NA	12.75	0.231 (0.024-2.235)	0.206

[a] survival rate at 10 days
[b] median survival time derived from Kaplan-Meier curves
[c] mean survival time derived from Kaplan-Meier curves
[d] hazard ratio (HR) derived from Cox regression

3.9. Plasma polyamine analysis in young and aged mice (Tables S32-S35)

Table S30. Summary of plasma polyamine contents by treatment for young female mice.

Polyamines by group	N	Mean	SD	p-value (vs Control)
**Putrescine**				
Control	3	18.06	1.97	
Sodium	5	14.69	0.79	0.99
DFMO	5	18.50	7.21	1
Sul	5	303.98	553.03	0.15
DFMO+Sul	5	36.01	43.45	0.93
**Spermidine**				
Control	3	3.78	2.45	
Sodium	5	0.04	0.08	0.13
DFMO	5	0.24	0.44	0.15
Sul	5	3.10	6.66	0.78
DFMO+Sul	5	0.18	0.24	0.15
**Spermine**				
Control	3	0.20	0.29	
Sodium	5	3.07	1.00	0.95
DFMO	5	4.70	4.68	0.92
Sul	5	61.37	122.56	0.16
DFMO+Sul	5	5.90	4.72	0.89
**Total polyamines**				
Control	3	22.05	4.39	
Sodium	5	17.80	1.58	0.99
DFMO	5	23.44	11.73	1
Sul	5	368.45	682.16	0.16
DFMO+Sul	5	42.09	47.44	0.93

Table S31. Summary of plasma polyamine contents by treatment for young male mice.

Polyamines by group	N	Mean	SD	p-value (vs Control)
**Putrescine**				
Control	4	18.27	3.48	
Sodium	5	2.18	0.91	<0.0001
DFMO	5	0.65	0.86	<0.0001
Sul	5	4.85	3.69	<0.0001
DFMO+Sul	5	2.38	1.30	<0.0001
**Spermidine**				
Control	4	1.47	0.63	
Sodium	5	2.87	2.16	0.17
DFMO	5	1.98	1.27	0.61
Sul	5	3.84	1.49	0.03
DFMO+Sul	5	3.73	1.14	0.03
**Spermine**				
Control	4	0.03	0.03	
Sodium	5	0.05	0.11	0.97
DFMO	5	0.46	0.62	0.3
Sul	5	2.00	0.85	<0.0001
DFMO+Sul	5	1.43	0.76	<0.01
**Total polyamines**				
Control	4	19.78	4.10	
Sodium	5	5.10	2.82	<0.0001
DFMO	5	3.08	2.67	<0.0001
Sul	5	10.68	4.12	<0.001
DFMO+Sul	5	7.54	2.23	<0.0001

Table S32. Summary of plasma polyamine contents by treatment for aged female mice.

Polyamines by group	N	Mean	SD	p-value (vs Control)
**Putrescine**				
Control	4	12.05	0.71	
DFMO	5	12.79	4.04	0.75
Sul	5	12.14	3.91	0.97
DFMO+Sul	5	13.63	3.07	0.49
**Spermidine**				
Control	4	1.42	1.00	
DFMO	5	2.01	1.54	0.59
Sul	5	3.02	2.42	0.15
DFMO+Sul	5	1.58	0.65	0.88
**Spermine**				
Control	4	0.00	0.00	
DFMO	5	0.12	0.15	0.17
Sul	5	0.06	0.14	0.48
DFMO+Sul	5	0.08	0.14	0.38
**Total polyamines**				
Control	4	13.47	0.46	
DFMO	5	14.92	5.65	0.65
Sul	5	15.22	5.67	0.58
DFMO+Sul	5	15.29	3.78	0.56

Table S33. Summary of plasma polyamine contents by treatment for aged male mice.

Polyamines by group	N	Mean	SD	p-value (vs Control)
**Putrescine**				
Control	3	14.11	1.81	
DFMO	4	12.96	2.51	0.63
Sul	4	20.76	4.93	0.02
DFMO+Sul	4	15.54	1.38	0.56
**Spermidine**				
Control	3	1.11	0.07	
DFMO	4	1.36	0.37	0.86
Sul	4	2.24	0.77	0.43
DFMO+Sul	4	3.51	3.34	0.11
**Spermine**				
Control	3	0.04	0.04	
DFMO	4	0.10	0.03	0.16
Sul	4	0.11	0.06	0.11
DFMO+Sul	4	0.06	0.07	0.62
**Total polyamines**				
Control	3	15.26	1.91	
DFMO	4	14.42	2.81	0.73
Sul	4	23.11	4.40	<0.01
DFMO+Sul	4	19.11	2.31	0.13

3.10. Summary of clinical scores (Tables S34-S37).

Table S34. Summary of clinical score by treatment for 6-week-old male mice.

Group	N	Mean	SD	p-value (vs. Control)
Control	3	8.33	0.58	
Sodium	5	6.00	3.39	0.19
Sul	5	9.40	1.14	0.54
DFMO	5	6.60	3.36	0.33
DFMO+Sul	5	9.60	0.89	0.47

Table S35. Summary of clinical score by treatment for 6-week-old female mice.

Group	N	Mean	SD	p-value (vs. Control)
Control	3	8.33	0.58	
Sodium	5	5.00	3.39	0.1
Sul	5	7.80	2.17	0.79
DFMO	5	7.60	3.78	0.71
DFMO+Sul	5	8.60	0.89	0.89

Table S36. Summary of clinical score by treatment for 58-week-old female mice.

Group	N	Mean	SD	p-value (vs. Control)
Control	4	5.0	1.83	
DFMO	5	4.6	4.16	0.85
Sul	5	7.0	3.94	0.351
DFMO+Sul	5	8.8	0.84	0.087

Table S37. Summary of clinical score by treatment for 58-week-old male mice.

Group	N	Mean	SD	p-value (vs Control)
Control	4	8.75	4.03	
DFMO	4	6.25	4.92	0.398
Sul	4	5.75	3.78	0.313
DFMO+Sul	4	4.25	3.20	0.14

3.11. Sulindac and sulindac metabolites analysis in young mice (Tables S38, S39).

Table S38. Summary of Sulindac metabolites by treatment for infected 6-week-old male mice.

Metabolites by group	N	Mean	SD	p-value (vs. Sul)
**Sulindac**				
Sul	5	11105.68	10442.08	
DFMO+Sul	5	12879.12	10466.35	0.8
**Sulindac Sulfide**				
Sul	5	4207.47	4363.02	
DFMO+Sul	5	2039.03	1525.07	0.32
**Sulindac Sulfone**				
Sul	5	17322.07	17829.82	
DFMO+Sul	5	19098.69	10452.43	0.85

Table S39. Summary of Sulindac metabolites by treatment infected 6-week-old female mice.

Metabolites by group	N	Mean	SD	p-value (vs. Sul)
**Sulindac**				
Sul	5	5152.45	3199.21	
DFMO+Sul	5	7625.51	6257.21	0.45
**Sulindac Sulfide**				
Sul	5	1784.43	734.56	
DFMO+Sul	5	8828.69	7984.65	0.09
**Sulindac Sulfone**				
Sul	5	5546.65	2929.11	
DFMO+Sul	5	7473.67	7494.03	0.61

3.12. Sulindac and sulindac metabolites in aged mice (Tables S40, S41)

Table S40. Summary of Sulindac metabolites by treatment for infected 58-week-old male mice.

Metabolites by group	N	Mean	SD	p-value (vs. Sul)
**Sulindac**				
DFMO+Sul	4	804.55	456.54	
Sul	4	2006.93	2103.03	0.31
**Sulindac Sulfide**				
DFMO+Sul	4	845.65	575.51	
Sul	4	2036.49	2137.77	0.32
**Sulindac Sulfone**				
DFMO+Sul	4	10204.07	6697.78	
Sul	4	12103.51	6579.06	0.7

Table S41. Summary of Sulindac metabolites by treatment for infected 58-week-old female mice.

Metabolites by group	N	Mean	SD	p-value (vs. Sul)
**Sulindac**				
DFMO+Sul	5	259.56	229.60	
Sul	5	2842.22	3854.47	0.17
**Sulindac Sulfide**				
DFMO+Sul	5	232.88	243.81	
Sul	5	2129.56	2562.26	0.14
**Sulindac Sulfone**				
DFMO+Sul	5	9176.47	6327.38	
Sul	5	20045.75	11026.01	0.09
